# VapA/Scs2 sustains polarized growth in *Aspergillus nidulans* by maintaining AP-2-mediated apical endocytosis

**DOI:** 10.15698/mic2026.02.868

**Published:** 2026-02-04

**Authors:** Xenia Georgiou, Sofia Politi, Sotiris Amillis, George Diallinas

**Affiliations:** 1 Department of Biology, National and Kapodistrian University of Athens, Panepistimioupolis, 15784 Athens, Greece; 2 Institute of Molecular Biology and Biotechnology, Foundation for Research and Technology, 70013 Heraklion, Greece

**Keywords:** fungi, traffic, sorting, secretion, ER-PM membrane contacts, membrane contact sites

## Abstract

Growth of filamentous fungi is highly polarized requiring the coordinated apical delivery of cell wall components and plasma membrane (PM) material, primarily lipids and proteins, to hyphal tips via conventional vesicular secretion. Fungal growth also requires the tight coordination of exocytosis (secretion) with endocytosis and recycling of proteins and lipids, which occurs in a defined region behind the growing tip known as the endocytic collar. Here, we genetically characterized proteins tentatively implicated in the formation of endoplasmic reticulum–plasma membrane (ER–PM) contact sites, including Scs2/VAP, tricalbins and Ist2 homologues, in *Aspergillus nidulans*. We showed that among these proteins, only the single Scs2/VapA homologue is essential for normal fungal growth, and this requirement is due to the critical role of VapA in maintaining the polarized localization of apical cargoes, such as the lipid flippases DnfA and DnfB or the SNARE protein SynA. In Δ *vapA* mutants, these cargoes lose their polarized localization, a phenotype that correlates with the mislocalization of the AP-2 cargo adaptor complex, which is essential for the endocytosis and recycling of apical membrane components. Further analysis provides evidence linking the defect in apical cargo endocytosis observed in Δ *vapA* mutants to altered membrane lipid partitioning, suggesting that VapA contributes to lipid domain organization critical for cargo recycling. Strikingly, deletion of VapA does not impair the localization or endocytosis of non-polarized (subapical) plasma membrane transporters, indicating that the trafficking and biogenesis of polarized (apical) versus non-polarized (subapical) cargoes are differentially dependent on membrane lipid composition and domain-specific organization.

## INTRODUCTION

In eukaryotic cells, the endoplasmic reticulum (ER) forms a dynamic membrane network that establishes close contacts with nearly all subcellular organelles and the plasma membrane (PM). These specialized regions, known as membrane contact sites (MCS), play a critical role in the non-vesicular transport of membrane lipids from the ER—the primary site of lipid synthesis—to other cellular compartments 1–5. Lipid transfer at MCS depends on specific lipid transfer proteins and calcium signaling, operating in concert with lipid metabolic pathways to preserve the unique lipid composition of each organelle membrane 6, 7. Among these, contacts between the cortical ER (cER) and the PM are particularly prominent, serving as key hubs for the transfer and distribution of phospholipids, sterols, and other lipid species to the PM 8–10. Proper lipid partitioning in both the PM and endomembranes is essential for the function, trafficking, and turnover of various membrane proteins, including enzymes, transporters, and receptors 11–13. Notably, ER-PM contact site proteins also influence ER-selective autophagy (ER-phagy) under conditions such as nutrient starvation or ER stress caused by misfolded protein accumulation, highlighting the broader significance of MCS in maintaining ER and cellular homeostasis 14.

**Figure 1:**
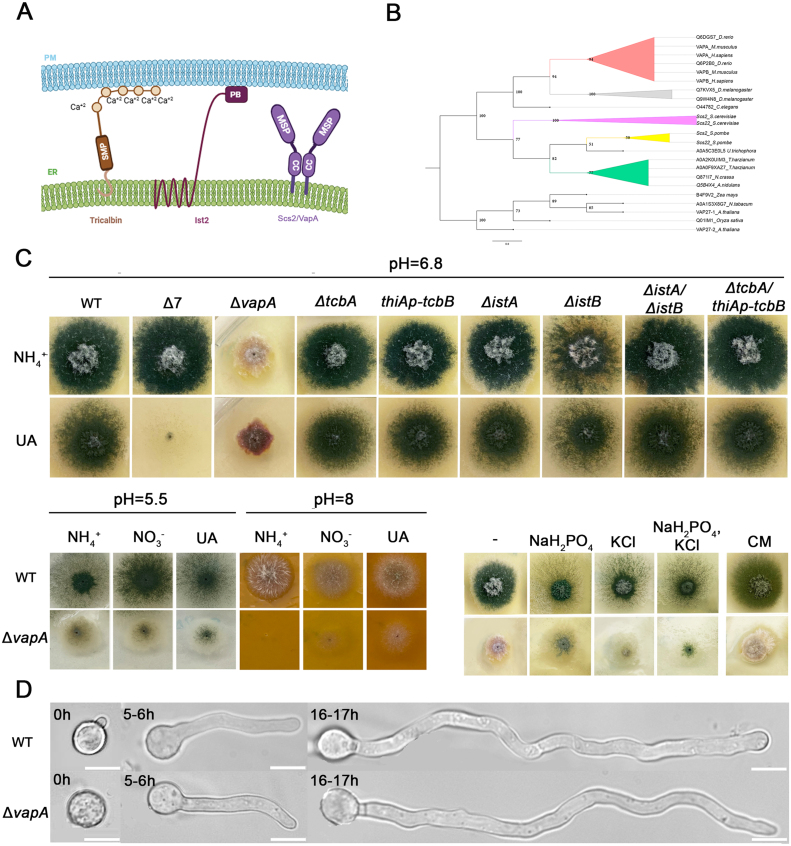
Phenotypic analysis of proteins tentatively associated to ER-PM contacts in *A. nidulans*. **(A) ** Schema illustrating three families of integral ER proteins that tether the cER to the PM, as characterized in yeasts (*S. cerevisiae* and *S. pombe*) and other eukaryotes: tricalbins, Ist2 and Scs2/VAP proteins. **(B)** Phylogenetic relationships of VapA/VAP homologues across model organisms. The tree was constructed using the maximum-likelihood method in IQ-TREE, with bootstrap support values shown in black. Organisms are grouped into three major phylogenetic clades: plants (root), fungi and metazoa. The fungal clade diverges from the metazoan lineage, with *S. cerevisiae* forming a distinct branch from the other fungal species. The dataset includes representative model organisms such as *Homo sapiens, Mus musculus, Arabidopsis thaliana, S. cerevisiae, S. pombe, Drosophila melanogaster* and *Caenorhabditis elegans.* The branch scale represents 0.8 substitutions per site, and branch lengths indicate the expected number of substitutions per site. Bootstrap support values were calculated using Ultrafast Bootstrap Approximation (1000 replicates) and SH-aLRT branch test (1000 replicates). **(C)** Growth phenotypes of single-gene deletions ( Δ vapA, Δ tcbA, Δ istA, Δ istB), double-gene deletions ( Δ istA/ Δ istB). Strains where tcbB is repressed via the thiA promoter (thiAp-tcbB and Δ tcbA/thiAp-tcbB) are also shown (Supplementary Figure S1A). A standard wild-type strain (wt) and a strain carrying multiple deletions in genes encoding purine/pyrimidine-related transporters are used as controls ( Δ *furD* Δ *furA* Δ *fcyB* Δ *uapA* Δ *uapC* Δ *azgA* Δ *cntA*, named Δ 7). In the upper panel, strains are grown on minimal media with selected nitrogen sources (ammonium/NH ${}_{4}^{+}$ or uric acid/UA), at pH = 6.8, 37 ${}^{\circ }$ C. Notice that solely Δ *vapA* exhibits dramatically reduced growth rate, conidiospore production (*i.e.*, absence of green color associated with conidiospores) and altered colony morphology. The lower panels depict growth phenotypes at pH = 5.5 or pH = 8, and on various hyperosmotic media (NaH ${}_{2}$ PO ${}_{4}$ , KCl or both) or standard Complete Medium (CM). Notice that the sporulation of Δ *vapA* is somehow enhanced at pH = 5.5, and with the addition of NaH ${}_{2}$ PO ${}_{4}$ and/or KCl in the minimal media. **(D)** Microscopy imaging of Δ *vapA,* compared to its wtstrain, at different development stages after germination (0–17 h or germination at 25 ${}^{\circ }$ C). Notice that Δ *vapA* exhibits apparently normal growth rate and germling formation in the time course of microscopy.

Proteins belonging to three distinct families have been shown to be essential elements of ER-PM contact formation and functioning in yeasts, plants and mammals. These proteins, schematically depicted in Figure 1**A,** are: the Ist2/TMEM16/anoctamin/protein family, which includes ion channels and phospholipid scramblases 8, 15, the tricalbin/E-Syts protein family 2, 8, 16, 17, and the VAMP (Vesicle Associated Membrane Protein)-associated protein family, known as VAPs 8, 18, 19. Here, for simplification, these three types of proteins are referred as Ist2, tricalbin and VAP.

In *Saccharomyces cerevisiae*, nearly half of the PM lies in close proximity (15–30 nm) to the cER, providing a valuable model for studying the formation, function, and physiological roles of ER–PM contact sites and their associated proteins 6, 8, 10, 20–22. Among these proteins, Ist2, embedded in the ER membrane via eight transmembrane segments, has a long cytoplasmic C-terminal tail that binds PM lipids, thereby tethering the ER to the PM 8, 15. Loss of Ist2 reduces cER levels, whereas its overexpression enhances ER–PM contacts 8, 23. The tricalbin proteins (Tcb1/2/3), yeast orthologs of mammalian extended synaptotagmins (E-Syts) and plant synaptotagmins (SYTs) 2, 16, 24, are anchored to the ER via a hairpin loop 16. These proteins contain a synaptotagmin-like, mitochondrial, and lipid-binding protein (SMP) domain that facilitates lipid binding and transfer 25–27, along with a variable number of C-terminal C2 domains that mediate phospholipid binding and ER–PM tethering 28, 29. Despite their established role as ER–PM tethers, deletion of tricalbins does not markedly affect cER abundance 8, 30, suggesting their primary function may lie in membrane remodeling or lipid dynamics rather than mechanical tethering. Lastly, Scs2 and Scs22, the yeast homologs of mammalian VAP proteins, are anchored to the ER via a single C-terminal transmembrane segment and contain an N-terminal major sperm protein (MSP) domain. These proteins tether the ER to the PM through interactions between the MSP domain and PM proteins bearing FFAT-like motifs 8, 18. Deletion of Scs2/22 leads to a significant reduction in cER levels 8. Each of these tether families contributes uniquely to cER morphology: Scs2/22 are associated with cER sheet formation, while tricalbins promote tubular cER structures at regions of high membrane curvature. While single deletions of Ist2, Scs2/22, or Tcb1/2/3 do not cause major cellular or phenotypic defects, simultaneous deletion of all three tethering systems significantly reduces ER–PM associations and contact site number. This strain, commonly referred to as ‘ Δ tether,’ exhibits markedly diminished cER and disrupted PM lipid homeostasis 8, 10. Notably, however, even in the Δ tether strain, residual ER–PM contacts persist, supporting that additional factors, as for example the integral ER membrane protein Ice2, contribute to ER–PM association.

ER–PM contact sites, and particularly the role of Scs2/VAP homologues, have also been investigated in the fission yeast *Schizosaccharomyces pombe* 31. One study demonstrated that ER–PM contacts serve as physical barriers to vesicular secretion, thereby confining exocytosis to the cell tips. This spatial restriction is essential for establishing polarized protein and lipid trafficking required for proper *S. pombe* growth 32. Another study revealed that eisosome-associated PM invaginations stabilize local ER–PM contacts through the interaction of Scs2 with Pil1, a core eisosomal component. This interaction influences the remodeling and dynamics of the cER 33. Additionally, the same research group showed that Scs2/VAP interactions with phospholipids regulate ER–PM contact formation, and provided evidence that direct binding between Scs2/VAP and Pma1, the major PM H ${}^{+}$ -ATPase, is crucial for maintaining pH homeostasis 34.

Similar to yeasts, filamentous fungi possess an extensive cER network that forms multiple contacts with the PM. To date, the only ER–PM tethering protein studied in a filamentous fungus is MoSCS2, a VAP homolog from the rice blast fungus *Magnaporthe oryzae.* Deletion of the MoSCS2 gene results in markedly reduced vegetative growth and conidiospore production, which is associated with decreased virulence. This is due to its role in conidia morphology, appressorium formation, and the inability to generate sufficient turgor pressure for host cell penetration. MoScs2 was also shown to be involved in regulating the cell wall and responding to endoplasmic reticulum stress 35.

To explore the functional significance of ER–PM contact sites in filamentous fungi, we identified and genetically characterized all *Aspergillus nidulans* proteins homologous to Snc2/VAP, Ist2, or tricalbins. We show that the sole Scs2/VAP homolog, VapA, is essential for growth, and provide evidence that this requirement stems from a failure to maintain the polarized localization of specific cargoes at the hyphal apex. This defect correlates with depolarization of the AP-2 adaptor complex, which is required for apical cargo endocytosis 36, and with disruptions in lipid homeostasis. Importantly, VapA deletion does not impair the trafficking, localization, or endocytosis of non-polarized nutrient transporters, suggesting that apical and subapical membrane cargoes depend differentially on partitioning into distinct lipid domains.

## RESULTS

### The VapA/Scs2 homologue is essential for sustaining polarized growth in *A. nidulans*

Most *Aspergillus* species possess homologues of ER–PM (ER–PM) tethering proteins with significant sequence similarity to those found in *S. cerevisiae* and *S. pombe*. These homologues, for example, share approximately 35% identity with *S. cerevisiae* and *S. pombe* proteins, 45% with other filamentous ascomycetes, 41% with basidiomycetes, and 29–30% with mammalian, insect, and plant VAPs. To identify *A. nidulans* homologues of Snc2/VAP, tricalbins, and Ist2 (Figure 1**A**), we performed BLASTp and BLASTx searches using the corresponding *S. cerevisiae* and *S. pombe* proteins as queries. This analysis revealed that *A. nidulans* encodes a single Scs2/VAP homologue (AN4406, hereafter VapA), two tricalbin homologues (AN9149, TcbA; and AN5624, TcbB), and two Ist2 homologues (AN2477**,**IstA**;**and AN7165, IstB). Using standard reverse genetics based on homologous recombination of DNA cassettes, we constructed null mutants for *vapA, tcbA, istA* and *istB* ( Δ *vapA*, Δ *tcbA*, Δ *istA*, and Δ *istB*, respectively). As we were unable to generate a knockout of tcbB, we instead generated a knockdown strain by replacing the native promoter with the tightly repressible *thiA* promoter (*thiAp-tcbB*) (Supplementary Figure S1B), as described previously 37, 38.

Phenotypic analysis of these mutants, summarized in Figure 1**C**, revealed that only the Δ *vapA* strain displayed a severe growth defect and a drastic reduction in conidiospore production. To assess potential redundancy, we also generated the double mutants Δ *istA*/ Δ *istB* and Δ *tcbA*/*thiAp-tcbB*. Neither of these combinations showed any apparent growth or morphological defects. Standard growth assays were performed on minimal medium (MM) at pH 6.8 and 37 ${}^{\circ }$ C. We further extended the growth analysis across a range of pH values, temperatures (25, 37, and 42 ${}^{\circ }$ C), and on complete medium (CM), to assess whether any of the mutants exhibited conditional growth defects or enhanced sporulation outside standard conditions (37 ${}^{\circ }$ C, pH 6.8). Under all conditions tested, the severe growth defect remained specific to the Δ *vapA* strain (Figure 1**C** and Supplementary Figure S1A, Supplementary Figure S2). Interestingly, the Δ *vapA* defect was modestly alleviated, particularly with respect to conidiospore production, at pH 5.5 or in salt-buffered media. Brightfield microscopy of Δ *vapA* and the WT strain during germination at 25 ${}^{\circ }$ C showed that Δ *vapA* exhibits normal germling formation and apical growth within the first 17 hours. This suggests that the severe growth impairment observed after 2–4 days of colony development arises from a cellular defect that accumulates at later stages of mycelial maturation and the onset of asexual sporulation. To investigate this, we measured the dry biomass of wtand Δ *vapA* strains, grown in liquid culture for 16 h, 24 h, and 48 h, at 25 ${}^{\circ }$ C (Supplementary Figure S1C). Consistent with our microscopic observations, no difference was detected at 16 h, whereas a significant reduction in Δ *vapA* biomass was observed at 24 h and 48 h. These results reinforce the hypothesis that VapA becomes important at later developmental stages.

Finally, Figure 1**B** presents a phylogenetic tree of VapA and its homologues across various model organisms. The topology reflects expected evolutionary relationships, with VapA clustering according to major taxonomic lineages such as fungi, mammals, insects, and plants.

### VapA is a cER protein

Since deletion of VapA, unlike any other putative ER–PM tether studied, resulted in a severe growth defect, we focused on characterizing its subcellular localization and functional role in more detail. To determine VapA localization *in vivo*, we generated GFP-tagged versions of the protein. Given that both N- and C-terminal regions of VAP proteins may influence their localization and function (see Figure 1**A**), we constructed three variants: GFP fused to the N-terminus (GFP-VapA), the C-terminus (VapA-GFP), and an internal insertion of GFP between the transmembrane (TM) domain, required for ER anchoring, and the functionally important coiled-coil domain (VapA-GFP-TM). To assess the role of ER anchoring in VapA function, we also generated a truncated version lacking the TM domain, with GFP fused to the C-terminus (VapA Δ TM-GFP). All constructs were integrated into the native (WT) *vapA* locus via homologous recombination, and transformants with correct gene replacement were verified by PCR (see Materials and Methodsfor details). In each case, expression was driven by the native *vapA* promoter to preserve physiological regulation.

**Figure 2:**
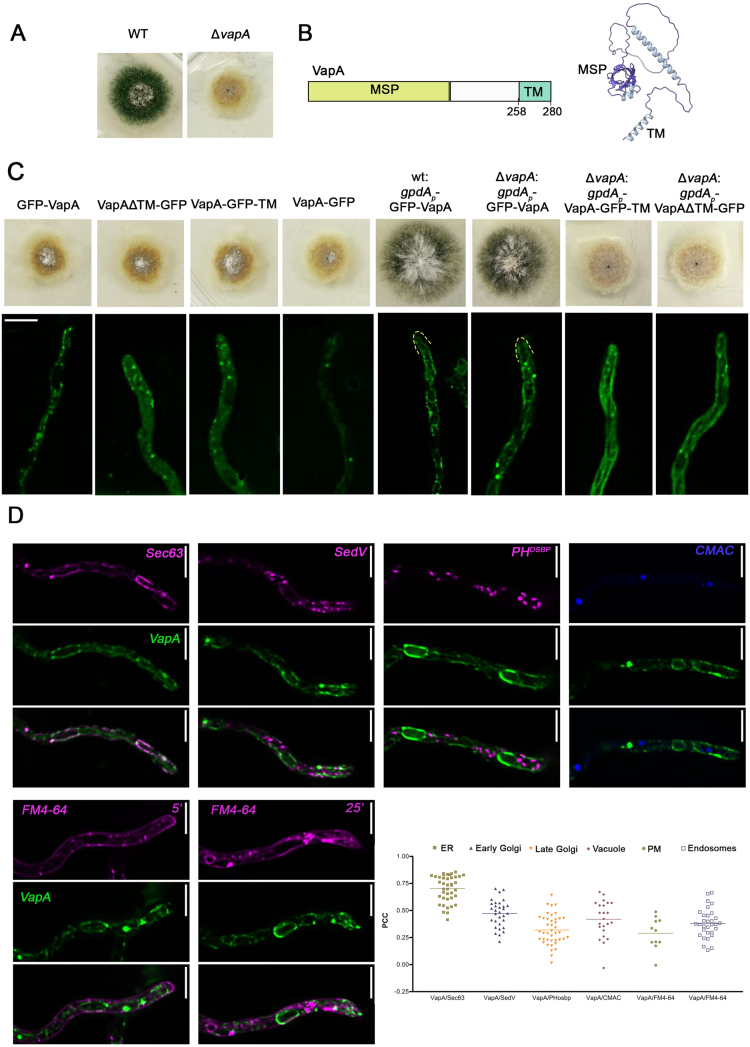
VapA is associated with the cER. **(A)** Growth tests of transformants expressing GFP-tagged versions of *vapA* gene. Notice that the *in-locus* substitution of the *vapA* gene with any of the GFP-tagged constructs made (GFP-VapA, VapA-GFP, VapA- Δ TM-GFP, VapA-GFP-TM) did not complement the defective growth phenotype of the original Δ *vapA* recipient strain. Nevertheless, overexpression of GFP-VapA under the *gpdA* strong promoter, unlike that of other constructs, rescued the Δ *vapA* phenotype, indicating that the N-terminal addition of GFP in VapA is functional. On the upper left, there is a cartoon depicting the different domains of VapA, and an alphafold-prediction of the protein (AN4406). Lower panels show maximal intensity projections of deconvolved snapshots indicating the subcellular localization of the corresponding versions of GFP-tagged VapA expressed under the native or the *gpdA* promoter. Notice that uniquely the overexpressed N-terminally tagged GFP-VapA labels a cortical and a perinuclear network resembling ER network. **(B)** Colocalization analysis of overexpressed GFP-VapA with standard markers of subcellular compartment (Sec63, SedV, PH ${}^{\mathrm{OSBP}}$ , CMAC and FM4-64). Maximal intensity projections of deconvolved z-stacks were used to quantify the co-localization of VapA with these compartment-specific protein markers in live cells. Pearson’s Correlation Coefficient (PCC) values were determined for VapA co-localization with Sec63 (ER; PCC = 0.70 ± 0.12, n = 40), SedV (early Golgi; PCC = 0.47 ± 0.12, n = 32), PH ${}^{\mathrm{OSBP}}$ (late Golgi; PCC = 0.32 ± 0.14, n = 41), FM4-64 for 5 min (endosomes; PCC = 0.38 ± 0.13, n = 31), FM4-64 for 20 min (plasma membrane; PCC = 0.29 ± 0.15, n = 11), and CMAC (vacuole; PCC = 0.42 ± 0.17, n = 23). Each dot represents the PCC of an individual cell Scale bars: 5 μ m.

All GFP-tagged VapA constructs failed to complement the growth defect of the Δ *vapA* null mutant when expressed under the native promoter (Figure 2**A**, middle panels). However, widefield fluorescence imaging revealed differences in localization depending on the site of GFP insertion (Figure 2**A**, lower panels). Notably, the N-terminally tagged GFP-VapA localized to a membranous network consistent with the endoplasmic reticulum (ER) morphology in *A. nidulans*, primarily labeling cortical membrane patches. In contrast, other constructs, such as VapA Δ TM-GFP and VapA-GFP-TM, displayed diffuse cytoplasmic fluorescence with punctate foci suggestive of membrane aggregates, while VapA-GFP exhibited only weak fluorescence.

Based on these observations, we hypothesized that the N-terminal GFP-VapA might localize correctly to the ER and retain partial functionality. To test this, and drawing on previous studies in fission yeast indicating that GFP-tagged VapA requires overexpression to restore function in deletion mutants 34, we constructed a version of GFP-VapA driven by the strong constitutive *gpdA* promoter (gpdAp-GFP-VapA). This construct was introduced into both WT and Δ *vapA* mutant strains. Expression of *gpdAp*-GFP-VapA successfully complemented the Δ *vapA* growth defect and had no dominant-negative effects in the WT background (Figure 2**A**, middle panels). Furthermore, this construct produced strong, ER-compatible fluorescence, consistent with the expected localization of a VAP protein. In contrast, overexpression of other constructs, including VapA Δ TM-GFP and VapA-GFP-TM, did not restore function and showed poorly defined, diffuse fluorescence patterns (Figure 2**B**, lower panels). Western blot analysis (Supplementary Figure S3) confirmed that only the N-terminally tagged GFP-VapA was stable. Other variants, especially those with internal GFP insertions such as VapA-GFP-TM, were unstable, probably due to rapid degradation under protein extraction conditions for the Western blot. Based on these results, the gpdAp-GFP-VapA strain was selected for all subsequent analyses.

To precisely identify the compartment labeled by *gpdAp-GFP-VapA* expression, we introduced fluorescent organelle markers into the strain via genetic crossing. These included Sec63 for the endoplasmic reticulum (ER), SedV for the ER-Golgi intermediate compartment (ERGIC) and early-Golgi, and PH ${}^{\mathrm{OSBP}}$ for the late-Golgi and the trans-Golgi network (TGN). Sec63, an essential subunit of the translocase complex, is a well-established ER marker in *A. nidulans* and other fungi, predominantly labeling the cER 39. SedV, a Q/t-SNARE syntaxin required for ER-to-Golgi vesicle transport, localizes to the ERGIC and early Golgi cisternae 38, 40, 41. PH ${}^{\mathrm{OSBP}}$ is a Pleckstrin Homology (PH) domain that specifically binds phosphatidylinositol-4-phosphate (PI4P), a lipid enriched in the late Golgi and TGN 38, 42, 43. To further characterize the labeled compartments, we also used the vacuolar lumen marker CMAC 44 and the endosomal/vacuolar membrane dye FM4-64 45. As shown in Figure 2**B**, GFP-VapA strongly colocalizes with the cER marker Sec63 (Pearson’s correlation coefficient, PCC = 0.70), consistent with its role as a VAP protein. Moderate colocalization was observed with the other markers (PCC = 0.29–0.47), reflecting the extensive interactions of the ER with other endomembrane compartments.

### VapA affects the recruitment of enzymes involved in lipid homeostasis at ER–PM contacts

**Figure 3:**
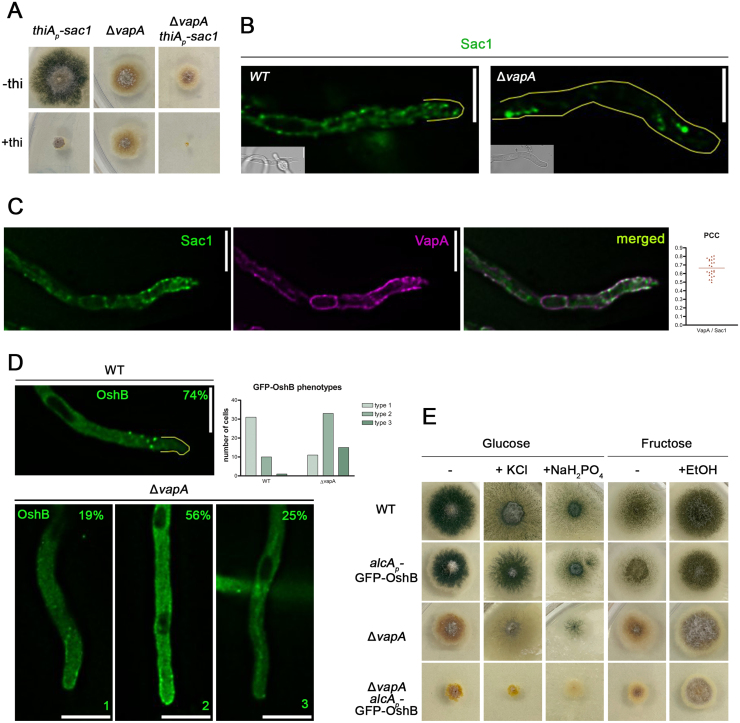
VapA affects the recruitment of Sac1 and OshB at ER–PM Contact Sites. **(A)** Growth tests of *thiA* ${}_{p}$ *-sac1*, Δ *vapA* and Δ *vapA thiA* ${}_{p}$ *-sac1,* in minimal medium in the presence or absence of thiamine. Notice, *thiA* ${}_{p}$ *-sac1* has significantly reduced colony size and sporulation, Δ *vapA thiA* ${}_{p}$ *-sac1* exhibits negative synthetic defect upon thiamine addition. **(B)** Maximal intensity projections of deconvolved snap shots showing Sac1-GFP in wtand Δ *vapA* and backgrounds. Notably, the localization of GFP-Sac1 is altered in Δ *vapA.* Sac1 is forming large intracellular aggregates, and displays reduced ER distribution. **(C)** Colocalization analysis of mCherry-VapA and Sac1-GFP. Maximal intensity projections of deconvolved z-stacks were used to quantify the co-localization of VapA with Sac1 in live cells. Pearson’s Correlation Coefficient (PCC) values were determined for VapA co-localization with Sac1 (PCC = 0.665 ± 0.096, n=23 ). Each dot represents the PCC of an individual cell. **(D)** Maximum intensity projections of deconvolved snap shots showing localization of *de novo* expressed GFP-OshB after 4 hours of induction with addition of 0.1% fructose and 0.4% v/v ethanol. In Δ *vapA*, three localization patterns were observed: 19% of cells had wt phenotype (type 1), 56% of cells displayed increased cytoplasmic and apical fluorescence (type 2), 25% of cells exhibited diffuse cytosolic haze (type 3). Altogether, GFP-OshB is mislocalized in 81% of Δ *vapA* cells and that percentage is significantly different than the one measured in wt(p < 0.05, p = 0.0241, n ${}_{\mathrm{wt}}$ = 41, n ${}_{\Delta vapA}$ = 59). **(E)** Growth tests of strains: wt, *alcA* ${}_{p}$ -GFP-OshB, Δ *vapA and* Δ *vapA alcA* ${}_{p}$ -GFP-OshB, in minimal media containing eighter glucose or fructose as carbon source, and the addition of salts (KCl, NaH ${}_{2}$ PO ${}_{4}$ ) or ethanol (EtOH), where indicated. The minus (–) symbol indicates that no salt or ethanol was added. Notice that repression of *oshB* (Glucose containing media) on Δ *vapA* background, results in reduced colony size and complete loss of sporulation. Induction of OshB expression (fructose and ethanol media), partially rescued colony morphology and sporulation of Δ *vapA.* Scale bars: 5 μ m.

In *S. cerevisiae*, the ER-resident phosphatase Sac1 dephosphorylates PI4P at the PM and interacts with the VAP homologues Scs2 and Scs22 to facilitate PI4P turnover specifically at ER–PM contact sites. Loss of Sac1 or its VAP tethers disrupts lipid homeostasis, resulting in PI4P accumulation and altered PM lipid composition 8, 46. To determine whether a similar mechanism exists in *A. nidulans*, we constructed a conditional mutant strain in which expression of *sac1* (AN3841) is controlled by the thiamine-repressible *thiA* promoter 47. As shown in Figure 3**A**, addition of thiamine led to strong repression of *sac1*, severely impairing colony formation and sporulation. These results confirm that *sac1* is essential for viability in *A. nidulans*. To investigate potential functional interactions between Sac1 and VAP proteins, we generated a double mutant strain, Δ *vapA thiAp-sac1*, and assessed its growth phenotype. This strain exhibited a complete absence of colony formation (Figure 3**A**), indicating a strong synthetic negative interaction between VapA and Sac1.

We next examined the subcellular localization of GFP-tagged Sac1 in both WT and Δ *vapA* backgrounds. In the WT background, GFP-Sac1 localized to a static, uniformly distributed network adjacent to the PM, consistent with ER localization. In contrast, in the Δ *vapA* mutant, Sac1 was mislocalized to large intracellular aggregates (Figure 3**B**). These results demonstrate that VapA is required for correct targeting of Sac1 to the ER and ER–PM contact sites.

To assess whether VapA and Sac1 are overlapping *in vivo*, we performed co-localization analysis. GFP-Sac1 exhibited strong spatial overlap with VapA at cER domains, yielding a Pearson’s correlation coefficient (PCC) of 0.665 (Figure 3**C**), indicating shared localization at ER–PM contact sites.

Oxysterol-binding homology (Osh) proteins are known regulators of lipid homeostasis and Sac1 activity at ER–PM contacts. In *S. cerevisiae*, Osh3, a PH domain–containing member of this family, functions as a PI4P sensor and Sac1 activator. Importantly, Osh3 recruitment to cER depends on Scs2/Scs22, highlighting a coordinated functional network between VAPs, Osh proteins, and Sac1 43, 46, 48. Based on the mislocalization of Sac1 in Δ *vapA* cells, we hypothesized that the localization of OshB, the *A. nidulans* orthologue of Osh3 49, might also depend on VapA. To test this, we crossed the Δ *vapA* mutant with a strain expressing GFP-OshB under the inducible *alcAp* promoter 49 and analyzed localization in WT and Δ *vapA* backgrounds. In a WT background, GFP-OshB showed diffuse cytoplasmic fluorescence and distinct cortical foci, particularly at the apical region of hyphae, consistent with its presence at ER–PM contact sites (Figure 3**D** and 49). In Δ *vapA* background, OshB localization was disrupted and could be classified into three distinct phenotypes: type 1 (WT-like localization, 19% of cells), type 2 (increased cytoplasmic and apical cortical fluorescence, 56%), and type 3 (diffuse cytosolic haze with no clear localization, 25%). Thus, in 81% of Δ *vapA* cells, OshB fails to properly localize to ER–PM contact sites.

To further investigate functional interactions between VapA and OshB, we analyzed the growth phenotype of the Δ *vapA alcAp-GFP-OshB* strain under conditions of *oshB* repression. As shown in Figure 3**E**, repression of *oshB* in a Δ *vapA* background led to reduced colony size and complete loss of sporulation. Conversely, overexpression of GFP-OshB, achieved by derepression in ethanol/fructose medium, partially restored both colony morphology and sporulation. These findings indicate that OshB overexpression can functionally compensate, at least in part, for the loss of VapA.

Collectively, our results demonstrate that VapA is required for the proper localization and function of both Sac1 and OshB at ER–PM contact sites in *A. nidulans*. These findings suggest a functional association between VapA, ORPs, and PI phosphatases within a conserved regulatory module controlling plasma membrane lipid homeostasis, although direct biochemical evidence will be required to substantiate this model.

### Deletion of VapA alters PH domain labeling and suggests a role in phospholipid partitioning

**Figure 4:**
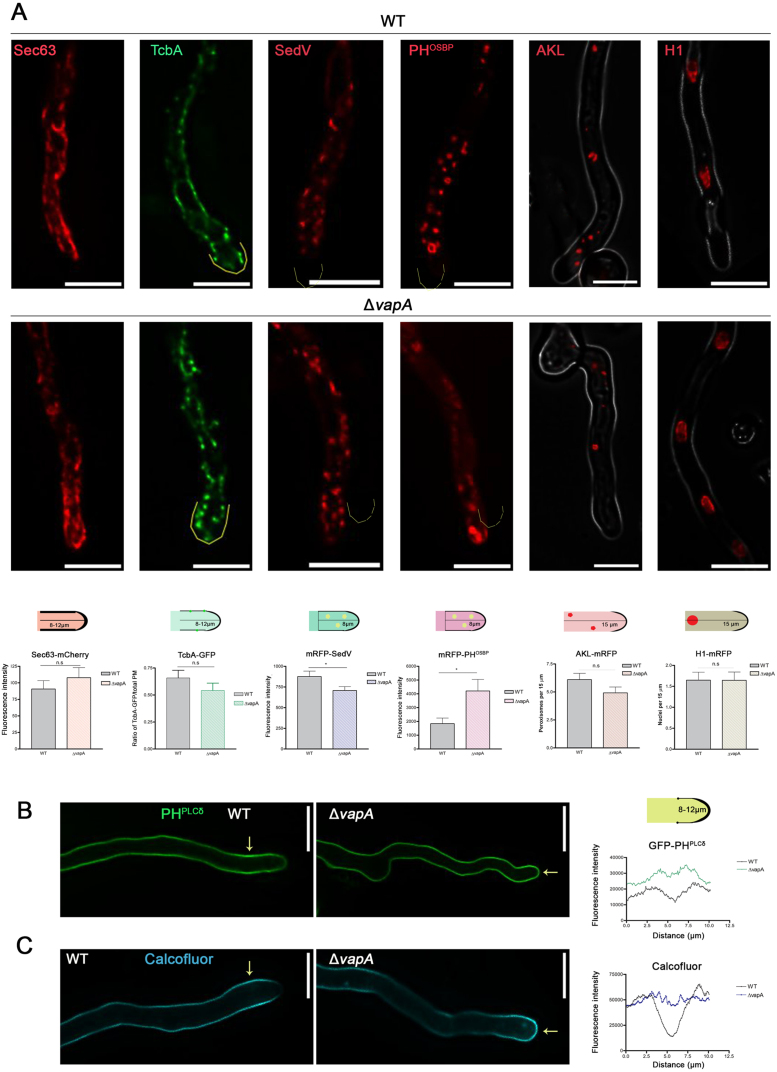
Deletion of *vapA* modifies labeling by PH and apical chitin deposition. **(A) ** Upper panel**:** Maximum intensity projections of deconvolved snap shots showing GFP/mRFP-tagged markers of the ER/cER (Sec63), cER-PM contacts (TcbA), ERGIC/early-Golgi (SedV), late-Golgi/TGN (PH ${}^{\mathrm{OSBP}}$ ), peroxisomes (mRFP-AKL) and nuclei (H1 histone) in WT and Δ *vapA* backgrounds. Lower panel: Bar plots for the analysis of Sec63, TcbA, SedV and PH ${}^{\mathrm{OSBP}}$ fluorescence respectively. For nuclei (H1-mRFP) and peroxisomes (AKL-mRFP), the number of fluorescent puncta was counted per 15 μ m hyphal segment. A cartoon on top of each graph illustrates the area of the hyphae that has been measured, error bars indicate the mean ± standard deviation for each group. For the statistical analysis of Sec63, TcbA, SedV, PH ${}^{\mathrm{OSBP}}$ , AKL and H1, an unpaired t-test was employed. For Sec63 and TcbA, the test revealed no significant difference in fluorescence intensity between WT and Δ *vapA* groups (WT ${}_{\mathrm{Sec63}}$ : Mean ± SEM = 90.63 ± 12.64 N = 13, Δ *vapA* ${}_{\mathrm{Sec63}}$ : Mean ± SEM = 107.7 ± 15.29 N = 17, t = 0.8263 df = 28, p = 0.4156), (WT ${}_{\mathrm{TcbA}}$ : Mean ± SEM = 0.6575 ± 0.07126 N = 17, Δ *vapA* ${}_{\mathrm{TcbA}}$ : Mean ± SEM = 0.5415 ± 0.06840 N = 20, t = 1.171 df = 35, p = 0.2495). For SedV, fluorescence was slightly but significantly reduced in Δ *vapA* (WT ${}_{\mathrm{SedV}}$ : Mean ± SEM = 875.4 ± 65.84 N = 24, Δ *vapA* ${}_{\mathrm{SedV}}$ : Mean ± SEM = 706.7 ± 47.21 N = 28, t = 2.123 df = 50, p = 0.0387). For PH ${}^{\mathrm{OSBP}}$ , fluorescence was significantly increased in Δ *vapA* (Unpaired t-test with Welch’s correction: WT ${}_{\mathrm{PH}}^{\mathrm{OSBP}}$ : Mean ± SEM = 1835 ± 409.7 N = 20, Δ *vapA* ${}_{\mathrm{PH}}^{\mathrm{OSBP}}$ : Mean ± SEM = 4207 ± 840.0 N = 20, t = 2.538 df = 27, p = 0.0172). For the number of peroxisomes and nuclei the test showed no significant difference (p > 0.05). **(B)** Epifluorescence microscopy showing the PM localization of the PI ${}_{\mathrm{4,5}}$ P-lipid marker PH ${}^{\mathrm{PLC}\delta }$ in WT and Δ *vapA* background. Notice that PH ${}^{\mathrm{PLC}\delta }$ distribution is altered in Δ *vapA*, specifically in the apical PM of hyphae (arrows indicate the difference between the WT and Δ *vapA*). The line plot of GFP-PH ${}^{\mathrm{PLC}\delta }$ fluorescence intensity along the PM of the hyphae tip ( μ m) displays the difference in intensity and distribution of the fluorescent signal (unpaired t-test with Welch’s correction: P<0.0001, hyphae measured N ${}_{\mathrm{WT}}$ = 27, N ${}_{\Delta \mathrm{vapA}}$ = 30). **(C)** Epifluorescence microscopy showing Calcofluor staining in WT and Δ *vapA* background. Calcofluor is a dye staining chitin, an essential component of the fungal cell of wall. Notice that, in Δ *vapA* there is increased chitin deposition in the extreme apex, rather than in the sub-apical collar. Arrows indicate the aforementioned regions in the mutant and wt strain respectively. The line plot of fluorescence intensity along the hyphae tip ( μ m) highlights the difference in calcofluor staining (unpaired t test with Welch’s correction: P < 0.0001, hyphae measured N ${}_{\mathrm{WT}}$ = 19, N ${}_{\Delta \mathrm{vapA}}$ = 19). Scale bars: 5 μ m. (More details on Materials and Methods section.)

We investigated whether deletion of *vapA* affects the subcellular organization and morphology of key organelles, which could underlie the observed growth defects. To this end, we introduced fluorescent molecular markers into the Δ *vapA* mutant background to label the cortical ER (cER; Sec63), ERGIC/early-Golgi (SedV), late-Golgi/trans-Golgi network (PH ${}^{\mathrm{OSBP}}$ ), nuclei (histone H1), and peroxisomes (mRFP-AKL) 39, 40, 42, 50–52. In addition to Sec63, we used TcbA-GFP, a tricalbin protein identified in this study, as a marker that more specifically labels cER–PM contact sites. mRFP-AKL is a chimeric construct that targets mRFP to peroxisomes 51, 52. As shown in Figure 4**A**, deletion of *vapA* has no apparent effect on the morphology of the cER, nuclei, or peroxisomes. Quantitative analysis of the early Golgi marker SedV revealed a reduction in fluorescence intensity in the Δ *vapA* strain compared with the WT, although no major structural alterations of the early Golgi were observed. However, we observed a significant 2.5-fold increase in PH ${}^{\mathrm{OSBP}}$ fluorescence at the apical region, suggesting enhanced interaction with phosphatidylinositol 4-phosphate (PI4P). This implies a local accumulation of PI4P at the late-Golgi/TGN in the absence of VapA.

To further explore phospholipid distribution at the PM, we examined a distinct PH domain protein, PH ${}^{\mathrm{PLC\delta}}$ , which binds specifically to phosphatidylinositol 4,5-bisphosphate (PIP2 or PI(4,5)P ${}_{2}$ ) 42, 53, 54. As shown in Figure 4**B**, loss of VapA resulted in a marked increase in PH ${}^{\mathrm{PLC\delta}}$ labeling at the apex of growing hyphae, indicating elevated levels of PI(4,5)P ${}_{2}$ in this region compared to the WT strain. Additionally, calcofluor white staining (Figure 4**C**) revealed increased fluorescence at the hyphal apex in the Δ *vapA* mutant, suggesting an increase in chitin deposition and altered cell wall composition.

Overall, these results indicate that VapA is required for proper phospholipid partitioning in both the late-Golgi and the apical PM. Its absence leads to the local accumulation of PI4P and PI(4,5)P ${}_{2}$ , as evidenced by enhanced binding of PH domain probes. These phospholipid changes are also associated with increased chitin deposition at the apex, further underscoring the role of VapA in maintaining phospholipid homeostasis, likely through its functional interactions with Sac1 and OshB.

### VapA is essential for polarized maintenance of growth-related cargoes via its essentiality for apical localization of the AP-2 adaptor complex

**Figure 5:**
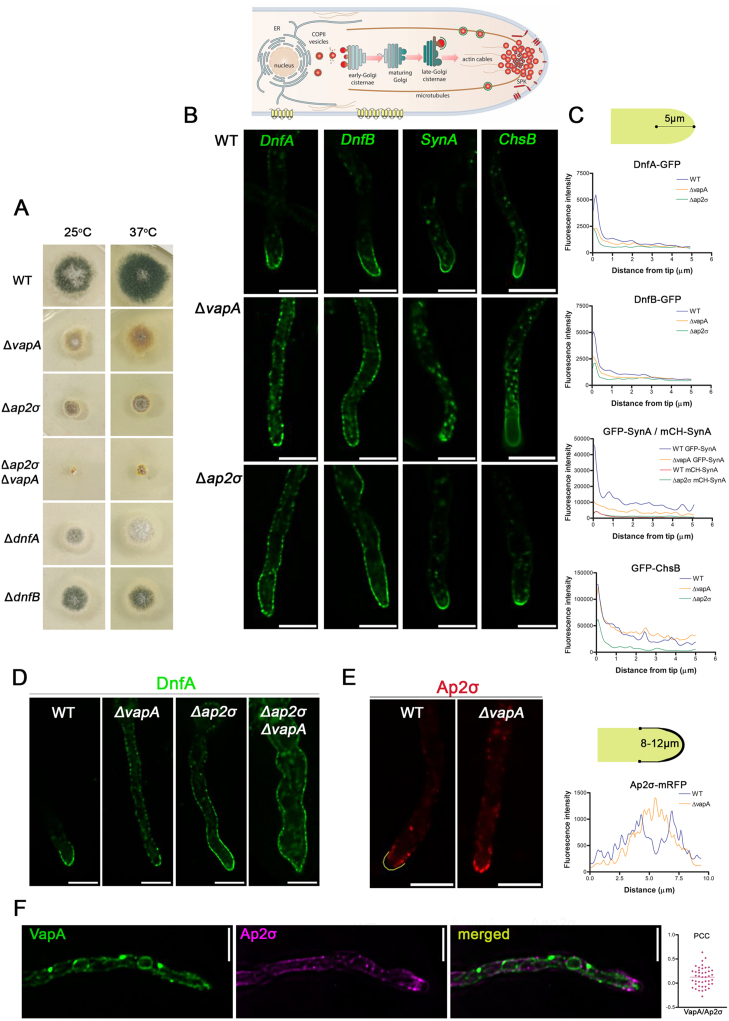
VapA is essential for maintaining the polarized localization of apical membrane proteins and sterol rich domains. **(A)** Cartoon depicting the polarized localization of apical markers and growth tests of Δ *vapA*, Δ *ap2* ${}^{\sigma }$ , Δ *vapA* Δ *ap2* ${}^{\sigma }$ , Δ *dnfA* and Δ *dnfB* null mutants and a WT control strain on standard synthetic minimal medium at 25 ${}^{\circ }$ and 37 ${}^{\circ }$ C. **(B)** Maximal intensity projections of deconvolved snap shots showing the localization of polarized membrane cargoes, DnfA, DnfB, SynA and ChsB in wt, Δ *vapA* and Δ *ap2* ${}^{\sigma }$ backgrounds. Notice that the apical markers DnfA and DnfB diffused in sub-apical segments of the PM in the Δ *vapA* mutant, rather similarly to Δ *ap2* ${}^{\sigma }$ *,* indicating a defect in polarized marker apical maintenance. SynA in Δ *vapA* was largely redistributed to intracellular aggregates, while it remained apically localized in Δ *ap2* $\${}^{\sigma }\$$ . ChsB localization appeared little affected in both mutants, yet Δ *ap2* $\${}^{\sigma }\$$ exhibited moderately lower fluorescence at the hyphae tip*.* For better comparison mCherry-SynA Δ *ap2* $\${}^{\sigma }\$$ is presented with green in this figure, but the original image and line plot are presented also in Supplementary Figure S4. Scale bars: 5 μ m. **(C)** Statistical analysis of cargo localization on the three strains. Line plots showing DnfA, DnfB, SynA, ChsB fluorescence intensity along the hyphal tip in wild-type (WT, blue), Δ *vapA* (orange) and Δ *ap2* ${}^{\sigma }$ (green) strains. The x -axis represents the distance from the hyphal tip ( μ m), and the y -axis represents fluorescence intensity. WT, Δ *vapA and* Δ *ap2* ${}^{\sigma }$ exhibit distinct fluorescence distributions along the hyphal axis. To assess spatial distribution differences, the fluorescence profiles were analyzed in 1 μ m distance bins from the hyphal tip (0–5 μ m). For each bin, pairwise comparisons were conducted and displayed in Data_Fig5. Cells measured: DnfA: N ${}_{\mathrm{WT}}$ = 18, N ${}_{\Delta \mathrm{vapA}}$ = 22, N ${}_{\Delta \mathrm{ap2}\sigma }$ = 17, DnfB: N ${}_{\mathrm{WT}}$ = 26, N ${}_{\Delta \mathrm{vapA}}$ = 28, N ${}_{\Delta \mathrm{ap2}\sigma }$ = 11, SynA: N ${}_{\mathrm{WT}}$ = 16, N ${}_{\Delta \mathrm{vapA}}$ = 18, N ${}_{\Delta \mathrm{ap2}\sigma }$ = 19, ChsB: N ${}_{\mathrm{WT}}$ = 23, N ${}_{\Delta \mathrm{vapA}}$ = 17, N ${}_{\Delta \mathrm{ap2}\sigma }$ = 14. Notice the clear depolarization of DnfA, DnfB and SynA fluorescence from the hyphae apex (0–1 μ M) in *vapA* null mutant, while in Δ *ap2* $\${}^{\sigma }\$$ significant depolarization was solely observed in DnfA and DnfB. **(D)** Snap shots showing the subcellular localization of DnfA in WT, Δ *vapA*, Δ *ap2* ${}^{\sigma }$ and Δ *vapA* Δ *ap2* ${}^{\sigma }$ backgrounds. In the double mutant Δ *vapA* Δ *ap2* ${}^{\sigma }$ DnfA displays a rather synthetic phenotype leading to increased mycelium width and increased accumulation of intracellular aggregates. **(E)** Epifluorescence microscopy showing the subcellular localization of Ap2 ${}^{\sigma }$ in wtand Δ *vapA* backgrounds. Notice that Ap2 ${}^{\sigma }$ seems immobilized on the extreme apex in Δ *vapA*, compared to the WT where Ap2 ${}^{\sigma }$ mostly marks the sub-apical collar. Line plot shows Ap2 ${}^{\sigma }$ fluorescence intensity around the hyphal tip, as illustrated in the cartoon, in wild-type (WT, blue) and Δ *vapA* (orange) strains. The x -axis represents the distance from the hyphal tip ( μ m) and the y -axis represents fluorescence intensity. The fluorescence profiles were analyzed in 1 μ m distance bins from 0–10 μ m. For each bin, pairwise comparisons were conducted and showed significant difference especially between 2–6 μ m. (P < 0.0001, N ${}_{\mathrm{WT}}$ = 17, N ${}_{\Delta \mathrm{vapA}}$ = 15). Notice the clear-cut difference at the area of the apex (3–7 μ M). **(F)** Snapshots showing the co-loacalization of Ap2 ${}^{\sigma }$ -mRFP and GFP-VapA, which reveals that Ap2 ${}^{\sigma }$ and VapA are in close proximity to each other but do not seem to colocalize. Dot plot displays the Pearson’s correlation coefficients (PCC) on y -axis (Mean ± SEM = 0.125 ± 0.03056, N = 43). Scale bars: 5 μ m.

The absence of VapA has a dramatic negative effect on hyphal growth, despite having no discernible impact on early developmental stages such as conidiospore germination or polarity establishment. This suggests that VapA may play a critical role in sustaining the maintenance of polarized localization of proteins required for the synthesis and/or deposition of new PM and cell wall materials at expanding hyphal tips. To explore this possibility, we examined the steady-state subcellular localization of several key apical proteins: the lipid flippases DnfA and DnfB 36, 55, chitin synthase ChsB 56, and the R/v-SNARE SynA 57. Deletion of *dnfA*, *dnfB,* or *chsB* 56 causes severe growth defects and a significant reduction in conidiospore production, phenotypes that closely resemble those observed in the Δ *vapA* mutant, particularly at 25 ${}^{\circ }$ C (see Figure 5**A**). Deletion of *synA* results in only mild growth impairment 57. In wild-type (wt) cells, these proteins are trafficked to the apical PM via the conventional Golgi- and exocyst-dependent pathway and remain restricted to the apical 2–3 μ m region through continuous AP-2-dependent endocytosis and recycling (see schematic in Figure 5**A**). Although AP-2 is typically considered a clathrin adaptor, we previously showed that in *A. nidulans*, it mediates apical cargo endocytosis independently of clathrin 36. Similar clathrin-independent roles for AP-2 have also been reported in mammalian central synapses 58. Notably, in *A. nidulans* deletion of any essential AP-2 subunit causes a severe growth defect, comparable to that seen in mutants lacking DnfA or DnfB 37 (see Figure 5**A**). Based on these findings, we hypothesized that VapA may be required to maintain the polarized apical localization of these essential cargoes. Because the localization of these proteins shows varying dependence on AP-2, we compared their distribution in Δ *vapA* and Δ *ap2* ${}^{\sigma }$ mutants and in a wtcontrol. Strains expressing DnfA, DnfB, ChsB, or SynA in wtand Δ *ap2* ${}^{\sigma }$ backgrounds were previously described 37, while the corresponding strains in the Δ *vapA* background were generated here through standard genetic crossing (see Materials and Methods).

Our results reveal that deletion of VapA leads to marked depolarization of DnfA, DnfB, and SynA (Figure 5**B** and Supplementary Figure S4). In the Δ *vapA* mutant, a significant proportion of these cargoes accumulates in subapical regions of the PM, often forming a gradient that extends several micrometers from the apical tip into the subapical PM. Noticeably, in Δ *vapA*, SynA is also localized to an increased number of cytoplasmic foci. Strikingly, the distribution patterns of DnfA and DnfB in Δ *vapA* closely resemble those observed in the Δ *ap2* ${}^{\sigma }$ mutant, where the cargoes are similarly mislocalized away from the apical region. Quantitative analysis of fluorescence intensity within 5 μ m of the apical tip supported these observations and confirmed a significant reduction in polarized cargo accumulation in the Δ *vapA* mutant (Figure 5**C**). These findings suggest that, as in Δ *ap2* ${}^{\sigma }$ , cargoes involved in lipid homeostasis in Δ *vapA* fail to undergo efficient apical endocytosis and recycling, resulting in their redistribution to subapical membrane domains. A similar phenotype is observed upon repression of key endocytic genes such as *slaB* and *myoV* (Supplementary figure S5). SlaB is an essential early endocytic adaptor that interacts with the AP-2/cargo complex at the PM, while MyoV is a type I myosin motor that functions during the later stages of endocytosis.

To investigate the functional relationship between VapA and the AP-2 complex, we also generated a double null mutant ( Δ *ap2* ${}^{\sigma }$ Δ *vapA*) via genetic crossing. We introduced a DnfA-GFP allele into this strain and compared its localization to that in each single mutant. As shown in Figure 5**D**, DnfA-GFP in the double mutant exhibited a non-polarized PM distribution similar to that of single *ap2* ${}^{\sigma }$ and *vapA* mutants. Interestingly, the Δ *vapA* Δ *ap2* ${}^{\sigma }$ strain also showed a heightened cytoplasmic signal associated with a membranous network, indicating a partially additive defect in DnfA localization when both VapA and AP-2 are absent. This cumulative disruption in DnfA trafficking correlated with reduced colony growth and pronounced morphological alterations in hyphae (Figures 5**A** and Figure 5**D**).

To further investigate the functional relationship between VapA and AP-2, we expressed a functional GFP-tagged version of AP-2 in the Δ *vapA* mutant. As shown in Figure 5**E**, the absence of VapA disrupts the normal localization of AP2-mRFP, leading to increased accumulation at the apex and a corresponding reduction in the subapical collar region (2–3 μ m from the tip), where active endocytosis and recycling of growth-related cargoes typically occur. Since AP-2 is recruited to the PM through a tripartite interaction with cargo proteins and specific phospholipids, particularly phosphatidylinositol 4,5-bisphosphate (PI(4,5)P ${}_{2}$ ), these findings suggest that VapA loss alters the organization of PM lipid domains, impairing AP-2 retention in the subapical collar and thus its ability to interact with apical cargoes. The additive effects observed in AP-2 and VapA double mutants further imply that VapA may also influence earlier trafficking events, likely involving anterograde cargo transport. Lastly, to examine whether VapA and AP-2 are spatially associated, we assessed their subcellular distribution by dual-fluorescence microscopy. As shown in Figure 5**F**, VapA and AP-2 do not colocalize, indicating that VapA likely affects AP-2 localization indirectly, most plausibly by modulating PM lipid composition.

### Further evidence supporting that VapA modifies membrane lipid partitioning in the PM

**Figure 6:**
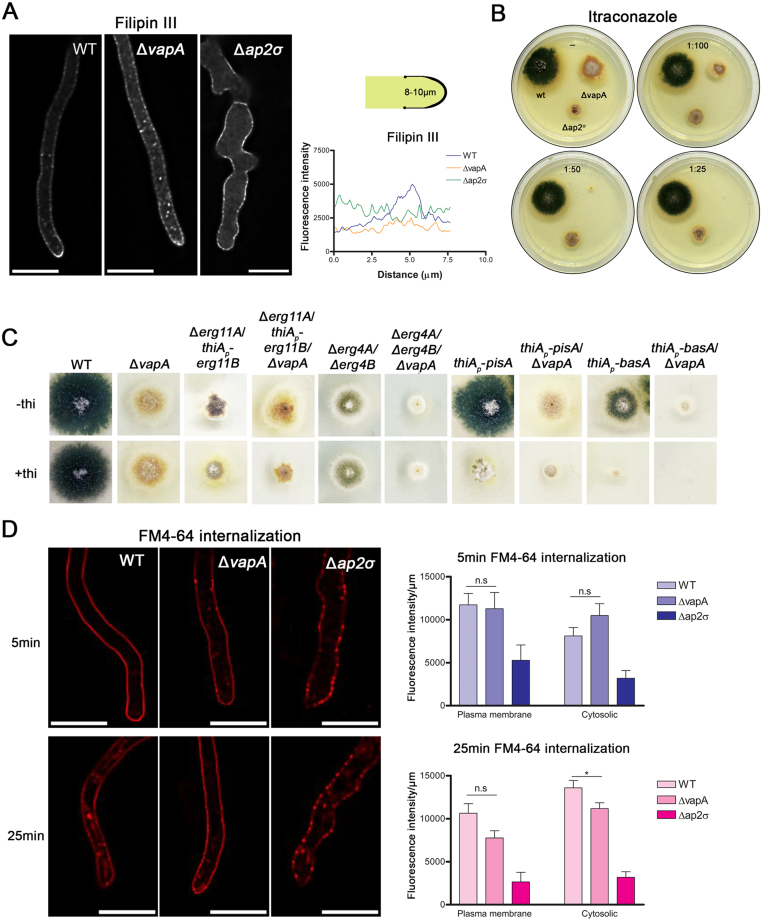
Evidence that VapA is crucial for proper membrane lipid homeostasis and partitioning to the PM. **(A)** Snap shots showing filipin III staining of ergosterol in WT, Δ *vapA*, Δ *ap2* ${}^{\sigma }$ . Notably, the null mutants exhibit distinct staining patterns, indicating altered ergosterol distribution at the PM and in particular reduced apical localization. This is statistically confirmed in a line plot, which represents filipin III fluorescence intensity along the hyphal tip ( μ m), as illustrated in the carton on the right. Specifically, to investigate spatial distribution differences, a Kruskal-Wallis test was first applied across the three strains, revealing significant global differences (P < 0.0001; H=152.3 , cells measured: N ${}_{\mathrm{WT}}$ = 20, N ${}_{\Delta \mathrm{vapA}}$ = 32, N ${}_{\Delta \mathrm{ap2}\sigma }$ = 18). The fluorescence profiles were analyzed in 1 μ m distance bins from 0–8 μ m. For each bin, pairwise comparisons were conducted and showed significant difference. (P < 0.0001). **(B)** Growth phenotypes of WT, Δ *vapA* and Δ *ap2* ${}^{\sigma }$ on minimal medium supplemented with subtoxic concentrations of itraconazole. Δ *vapA* displays heightened sensitivity to itraconazole even at low concentrations, in contrast to WT and Δ *ap2* $\${}^{\sigma }\$$ , which exhibit normal or near-normal growth. **(C)** Growth test of various strains repressed for genes involved in lipid biosynthetic pathways, on minimal medium in the presence or absence of thiamine. Strains include WT, Δ *vapA,* Δ *erg11A/thiAp-erg11B,* Δ *erg11A/thiAp-erg11B/* Δ *vapA,* Δ *erg4A/* Δ *erg4B,* Δ *erg4A/* Δ *erg4B/* Δ *vapA, thiAp-pisA, thiAp-pisA/* Δ *vapA, thiAp-basA, thiAp-basA/* Δ *vapA.* Notably, the combination of Δ *vapA* with repression or deletion of genes involved in lipid biosynthesis - *erg11A/B, erg4A/B,* and *pisA* - results in a synthetic growth defect, indicating potential genetic interactions between VapA and key lipid metabolic pathways. **(D)** Maximal intensity projections of deconvolved snap shots showing FM4-64 distribution in WT, Δ *vapA* and Δ *ap2* ${}^{\sigma }$ at 5 min and 25 min post-staining. Unpaired t-test between WT and Δ *vapA*, revealed a statistically significant decrease in cytosolic fluorescence intensity in Δ *vapA* at 25 minutes (p = 0.0308, t = 2.201 df = 75, N ${}_{\mathrm{WT}}$ = 38, N ${}_{\Delta \mathrm{vapA}}$ = 39), indicating delayed or altered FM4-64 internalization. Scale bars: 5 μ m. For additional interpretation see main relevant text.

As several apical markers are thought to partition within sterol-rich membrane domains in fungi 59, we investigated whether ergosterol distribution is altered in the Δ *vapA* and Δ *ap2* ${}^{\sigma }$ mutants compared to the wt strain, which typically exhibits enriched filipin staining at hyphal tips 60. Filipin dye staining revealed that deletion of either VapA or AP-2 resulted in a pronounced depolarization of filipin accumulation, with staining appearing uniformly distributed along the PM rather than concentrated at the tips (Figure 6**A**). This suggests that ergosterol partitioning is disrupted in both mutants, leading to a loss of polarized membrane organization. Given this apparent alteration in ergosterol localization, we next assessed whether the Δ *vapA* mutant displayed altered sensitivity to ergosterol-targeting antifungal agents relative to the wtand Δ *ap2* ${}^{\sigma }$ strains. As shown in Figure 6**B**, the Δ *vapA* mutant was significantly more sensitive to subtoxic concentrations of itraconazole than either the wtor Δ *ap2* ${}^{\sigma }$ strain. This finding supports the idea that VapA contributes to processes upstream of endocytic cargo trafficking, likely including the regulation of membrane sterol composition.

To further probe the role of VapA in membrane lipid homeostasis, we tested for synthetic growth defects in double mutants combining Δ *vapA* with alleles affecting major membrane lipid biosynthetic pathways. These included: (a) double knockout or knockdown of mutants in ergosterol biosynthesis ( Δ *erg11*A/*thiAp-erg11B* and Δ *erg4A*/ Δ *erg4B*), (b) a knockdown mutant of phosphatidylinositol synthase (*thiAp-pisA*), and (c) a knockdown mutant of sphingolipid C4 hydroxylase (*thiAp-basA*), as previously described 11. In all cases, the resulting double mutants exhibited exacerbated growth defects compared to their respective single mutants, indicating that loss of VapA imposes a synthetic burden on strains with impaired lipid biosynthesis (Figure 6**C**).

We also examined the impact of VapA on endocytosis using FM4-64, a lipophilic fluorescent dye that initially incorporates into the PM and is subsequently internalized via bulk endocytosis. FM4-64 internalization is sensitive to PM fluidity and lipid composition. In the Δ *vapA* mutant, initial PM labeling was comparable to the wtafter 5 minutes; however, the dye failed to label internal endosomal membranes even after 25 minutes, a time point at which robust internalization is normally observed in the wt(Figure 6**D**). This indicates that PM lipid composition and fluidity are significantly altered in the absence of VapA. Interestingly, FM4-64 uptake was also delayed in the Δ *ap2* ${}^{\sigma }$ mutant. Moreover, at the 5-minute time point, FM4-64 labeling in Δ *ap2* ${}^{\sigma }$ was already distinct from both wtand Δ vapA, displaying punctate cortical staining rather than a uniform PM signal, further supporting a functional link between VapA and the AP-2 complex.

### VapA is crucial for polarized recruiting of the endocytic machinery at the apical region

Since VapA was shown to influence apical cargo endocytosis by modulating AP-2, we investigated whether it also affects additional key components of the endocytic machinery that function downstream of AP-2, such as SlaB ${}^{\mathrm{Sla2/End4}}$ , SagA ${}^{\mathrm{End3}}$ , AbpA ${}^{\mathrm{Abp1}}$ or MyoA ${}^{\mathrm{Myo5}}$ 36, 61. As previously mentioned, SlaB is an adaptor protein that, together with Ent1 and Ent2, forms a midcoat complex following AP-2’s interaction with cargo. SagA is an EH domain-containing protein that forms a complex with Sla1 and Pan1, contributing to the next layer of the endocytic coat and playing a central role in actin organization. AbpA is an essential actin-binding protein, while MyoA represents the major class I myosin motor involved in the late stages of actin-driven endocytosis, functioning independently of earlier endocytic steps 62.

**Figure 7:**
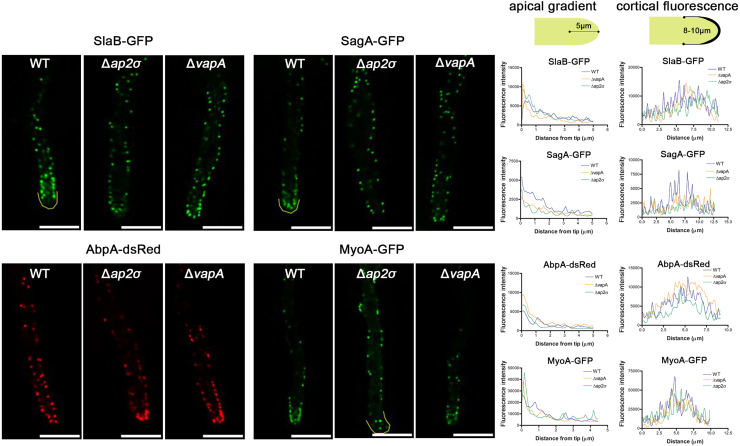
VapA is crucial for the proper functioning of the endocytic machinery at the apical region. **(A)** Epifluorescence microscopy showing the subcellular localization of endocytic markers SlaB, SagA, AbpA and MyoA in WT, Δ *ap2* ${}^{\sigma }$ or Δ *vapA*, backgrounds. SlaB and SagA lost their apical localization in Δ *vapA* and Δ *ap2* $\${}^{\sigma }\$$ . AbpA, which marks PM-associated actin patches, showed increased localization at hyphae tips in Δ *vapA* and Δ *ap2* ${}^{\sigma }$ *,* compared to WT. MyoA seemed little affected in Δ *vapA,* while it showed increased localization in non-polarized regions of the hyphae in Δ *ap2* $\${}^{\sigma }\$$ *.* Line plots quantifying AbpA, SlaB, SagA, MyoA fluorescence intensity along the hyphal tip (as illustrated in the cartoon) in wild-type (WT, blue) and Δ *vapA* (orange) and Δ *ap2* ${}^{\sigma }$ (green) strains. The x -axis represents the distance from the hyphal tip ( μ m), and the y -axis represents fluorescence intensity. To further access different labelling patterns, Curve plots displaying fluorescence intensity around the hyphal tip (as illustrated in the cartoon). The fluorescence profiles were analyzed in 1 μ m distance bins, 0–5 μ m for line plots, 0–12 μ m for curve plots. For each bin, pairwise comparisons were conducted and displayed in Data_Fig7. Scale bars: 5 μ m.

These endocytic proteins typically are accumulated in a polarized manner at the subapical collar of *A. nidulans* and other filamentous fungi, a region characterized by high levels of constitutive endocytosis 36, 37, 63–68. To assess whether VapA affects their localization, we generated functional fluorescent chimeras of SlaB-GFP, SagA-GFP, AbpA-mRFP, and MyoA-GFP (Materials and Methods), and introduced them into the Δ *vapA* strain. We then compared their steady-state subcellular localization to that observed in a WT strain and in the Δ *ap2* ${}^{\sigma }$ mutant background (Figure 7). Although some minor differences were observed among individual proteins, both imaging and quantitative analyses revealed a consistent trend: deletion of *vapA* or *ap2* σ reduced the polarization of endocytic factors at the hyphal tip. In the Δ *vapA* and Δ *ap2* σ strains, SlaB-GFP had increased accumulation at the apex (0–1 μ m), was reduced in the subapical collar region (1–3 μ m from the apex), and appeared diffusely distributed along the hyphae, in contrast to the WT, where SlaB-GFP was predominantly confined to the subapical collar. Similarly, SagA-GFP displayed a scattered distribution in both mutants, rather than the distinct apical localization observed in the WT. AbpA-dsRed showed increased signal at the apex (0–1 μ m) in Δ *vapA* and Δ *ap2* σ , resembling the apical overaccumulation previously seen for Ap2 σ -mRFP in Δ *vapA* (Figure 5E). MyoA-GFP localization was altered in Δ ap2 σ , where it became depolarized, yet remained apically enriched in Δ *vapA*, similar to the WT. Altogether, these findings indicate that the absence of VapA disrupts AP-2 sub-apical localization, which in turn impairs the proper spatial organization of early endocytic factors acting downstream of AP-2.

### VapA has a minor role in the secretion of apical cargoes

**Figure 8:**
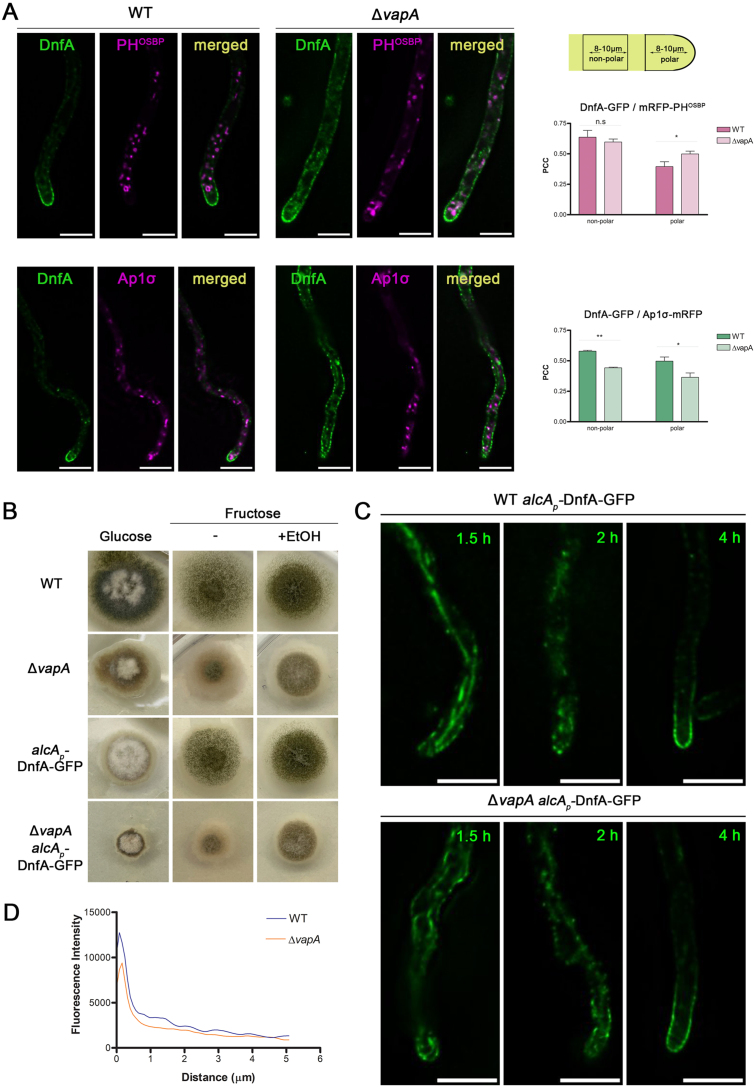
VapA has a minor role in the secretion of apical cargoes. **(A)** Epifluorescence microscopy showing the co-localization of DnfA with PH ${}^{\mathrm{OSBP}}$ and Ap1 ${}^{\sigma }$ in wtand Δ *vapA.* Co-localization was assessed in both apical (polarized) and subapical (non-polarized) regions of hyphae, as indicated in the accompanying scheme. DnfA exhibited significantly reduced overlap with Ap1 σ in Δ *vapA* compared to wtin both regions measured (non-polar: PCC ${}_{\mathrm{wt}}$ = 0.579, PCC ${}_{\Delta \mathrm{vapA}}$ = 0.442, p ${}_{\mathrm{-value}}$ : 0.0055, N ${}_{\mathrm{WT}}$ = 20, N ${}_{\Delta \mathrm{vapA}}$ = 32, polar: PCC ${}_{\mathrm{wt}}$ = 0.4972, PCC ${}_{\Delta \mathrm{vapA}}$ = 0.36480, p ${}_{\mathrm{-value}}$ : 0.0244, N ${}_{\mathrm{WT}}$ = 16, N ${}_{\Delta \mathrm{vapA}}$ = 32) and increased co-localization with the late Golgi marker PH ${}^{\mathrm{OSBP}}$ , specifically at the hypha tip (polar: PCC ${}_{\mathrm{wt}}$ = 0.395, PCC ${}_{\Delta \mathrm{vapA}}$ = 0.499, p ${}_{\mathrm{-value}}$ : 0.0268). This result might indicate a defect or delay in packaging of DnfA in post-Golgi AP-1 secretory vesicles. Accompanying bar plots display Pearson’s correlation coefficients (PCC) on the y -axis, separated by polarized and non-polarized measurements for WT and Δ *vapA.* Scale bars: 5 μ m. **(B)** Growth tests of strains: wt, Δ *vapA*, alcAp-DnfA-GFP and Δ *vapA* alcAp-DnfA-GFP, in minimal media containing eighter glucose or fructose as carbon source, and addition of ethanol (EtOH), where indicated. The minus (–) symbol indicates that no ethanol was added. Notice that, alcAp-DnfA-GFP exhibits a defective growth phenotype, similar to Δ *dnfA* (Figure 5A)*,* when repressed in the presence of glucose. **(C)** Maximum intensity projections of deconvolved snap shots showing localization of *de novo* expressed DnfA-GFP after 1.5, 2 or 4 hours of induction with addition of 0.1% fructose and 0.4% v/v ethanol in the media. Notice that, DnfA-GFP is localized at the PM of tip at 4 h, in a polarized manner, in both strains, thus indicating that the exocytosis to the apical region of the hyphae is practically unaffected in the Δ *vapA* null mutant. **(D)** Line plot showing DnfA fluorescence intensity along the hyphal tip in wtalcAp-DnfA-GFP (WT, blue) and Δ *vapA* alcAp-DnfA-GFP (orange). The x -axis represents the distance from the hyphal tip ( μ m), and the y -axis represents fluorescence intensity. For this quantification, we measured DnfA-GFP fluorescence after 4 hours of induction with addition of 0.1% fructose and 0.4% v/v ethanol. To assess spatial distribution differences, the fluorescence profiles were analyzed in 1 μ m distance bins from the hyphal tip (0–5 μ m). For each bin, pairwise comparisons were conducted, and found that there was no significant difference (p > 0.05) in the fluorescence intensity between wtand Δ *vapA.* Cells measured: N ${}_{\mathrm{WT}}$ = 24, N ${}_{\Delta \mathrm{vapA}}$ = 26.

Apart from its major role in apical cargo endocytosis and recycling, the lack of VapA might also lead to a minor defect in cargo secretion at the apical region. This was suggested by the observation that in several hyphae of the Δ *vapA* or Δ *vapA* Δ *ap2* ${}^{\sigma }$ mutants we could also detect an increase in labeling of cytoplasmic membrane-like structures, mostly detected by DnfA-GFP (see Figure 5**B** or **5D**). These cytoplasmic structures might correspond to the presence of cargoes in secretory compartments (e.g., *late*-Golgi/TGN or *post*-Golgi secretory carriers) that fail to reach the apical segment or are delayed in their secretion. To investigate this possibility, we recorded the colocalization of DnfA-GFP with fluorescent markers of the *late*-Golgi/TGN (mRFP-PH ${}^{\mathrm{OSBP}}$ ) and *post*-Golgi carriers (AP-1 ${}^{\sigma }$ -mRFP) 37 in wt and Δ *vapA* backgrounds. Figure 8**A** shows that in the absence of *vapA*, DnfA-GFP appears to have increased colocalization with late-Golgi (mRFP-PH ${}^{\mathrm{OSBP}}$ ) and moderately reduced colocalization with AP-1 ${}^{\sigma }$ -mRFP. These finding suggest that VapA might have a minor role in the dynamics of cargo secretion.

To address the effect of VapA on apical cargo secretion more directly, we performed an experiment which allowed us to follow the dynamics of DnfA exocytosis to the apical region. For this, we constructed new isogenic *vapA* ${}^{+}$ (wt) and Δ *vapA* strains in which the native *dnfA* promoter is replaced, in locus, by the regulatable promoter *alcA* ${}_{p}$ . This promoter is tightly repressed in the presence of glucose, but is derepressed when shifted to fructose and its activity is further enhanced when ethanol is also added in the medium. Notice that absence of DnfA expression leads to significant reduction in growth and colony morphology (Figure 5**A**). As shown in the growth tests of Figure 8**B**, in MM supplemented with fructose and ethanol, the strains expressing *alcA* ${}_{p}$ *-*DnfA-GFP grow similarly to the respective strains expressing DnfA from its native promoter. This confirmed that DnfA expression via *alcA* ${}_{p}$ in derepressing-inducing conditions is physiologically relevant. We used these strains to follow, *ab initio*, the trafficking of neosynthesized DnfA in *vapA* ${}^{+}$ and Δ *vapA* backgrounds (Figure 8**C**). Cells were grown overnight in glucose and next day shifted to fructose/ethanol medium. The fluorescent signal started becoming visible, in both strains, after 60–75 min and labeled the ER-like network at 90–120 min (Figure 8**C**). At 4 h, the signal accumulated, in a polarized manner, to the PM of the apical region, in both strains, strongly indicating that the exocytosis to the apical region is practically unaffected in the Δ *vapA* null mutant. The only difference in the distribution of DnfA in the two strains was that in Δ *vapA* the signal extended to a larger part of the PM towards the subapical segments (Figure 8**C**), yet this slight depolarization proved to be insignificant (Figure 8**D**). Thus, we concluded that absence of VapA leads to no significant effect on exocytosis, acting principally on the endocytic recycling of cargos at the apex.

### VapA is redundant for the localization of transporters and other non-apical cargoes

All evidence obtained strongly suggests that in the Δ *vapA* mutant, polarized apical cargoes cannot undergo proper AP-2–dependent endocytosis. Additionally, the deletion of VapA appears to reduce the steady-state accumulation of key endocytic factors at the subapical collar region. However, in *A. nidulans,* a distinct endocytic mechanism operates at the subapical regions of the PM, specifically involved in the turnover of several nutrient transporters. These transporters are localized in an antipolar manner, *i.e.*, they are absent from the apical tips 36, 69. Their endocytosis occurs in response to specific physiological or stress signals and, while clathrin-dependent, is surprisingly independent of AP-2 36, 96. Notably, these transporters also translocate to the PM via a Golgi-independent route, distinct from the conventional secretion pathway used by apical cargoes 38, 57, 69, 70. This unconventional trafficking route is also utilized by other non-polarized cargoes, such as the proton pump ATPase PmaA and PalI, a component of the alkaline pH-sensing system 69. Given these distinct mechanisms, we sought to determine whether VapA influences the localization and/or endocytosis of nutrient transporters and other non-polarized cargoes.

**Figure 9:**
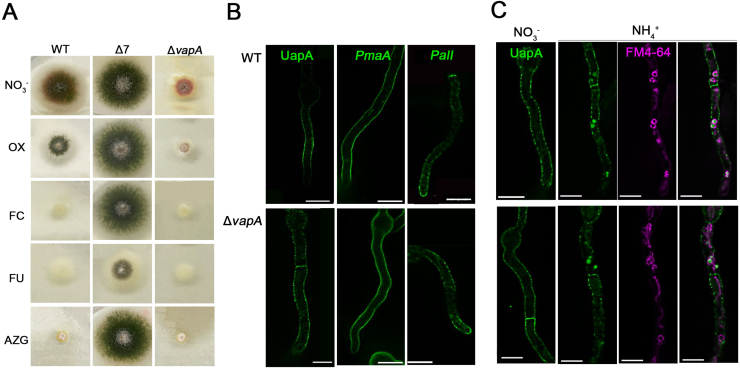
VapA is redundant for the localization and endocytosis of transporters and other non-apical cargoes. **(A)** Growth tests of Δ *vapA* in toxic nucleobase analogues. The toxic analogues used, oxypurinol (OX), 5-fluorocytosine (FC), 5-fluorouracil (FU), 8-azaguanine (AZG), were added in media containing nitrate as a nitrogen source, at 37 ${}^{\circ }$ C. A wtstrain was used as a negative control and Δ 7 as a positive control. These analogues are incorporated in the cells by several different transporters (OX by UapA, FC by FcyB, FU by FurD and AZG by AzgA; The Δ 7 strain lacks these transporters, as well as   other secondary related transporters, and is thus resistant to the toxic nucleobase analogues; see ref). Δ *vapA* shows the same phenotype as the wtstrain in the presence of toxic nucleobases, strongly suggesting that all major nucleobase transporters are functionally localized to the PM. **(B)** Maximal intensity projections of deconvolved snap shots showing the localization of non-polarized membrane cargoes, UapA, PmaA (PM proton pump ATPase) and PalI (pH sensing component of the PM) in wtand Δ *vapA.* The results confirm that all these non-polarized transmembrane cargoes have normal PM distribution in Δ *vapA.***(C)** Epifluorescence microscopy showing the endocytosis of UapA in wtand Δ *vapA.* UapA transporter is derepressed overnight in the presence of nitrate, then incubated with ammonium for 2 h, to trigger its internalization and subsequent vacuolar degradation. FM4-64 was used to monitor endosomal and vacuolar compartment. The results showed that UapA is localized in vacuoles in both strains (yellow arrows) after addition of NH4 ${}^{+}$ , indicating that transporter endocytosis and turnover is unaffected in Δ *vapA.* Scale bars: 5 μ m.

To assess whether VapA deletion affects transporter activity by impairing trafficking to the PM, we performed growth assays using toxic analogues of transporter substrates. These analogues, oxypurinol (OX), 5-fluorouracil (FU), 5-fluorocytosine (FC), and 8-azaguanine (AZG), are taken up by the UapA/UapC, FurD, FcyB, and AzgA transporters, respectively 71. Wild-type *A. nidulans* is sensitive to these compounds due to efficient uptake, whereas a strain carrying deletions of seven relevant transporter genes ( Δ 7) shows resistance 72, 73, as demonstrated in Figure 9**A**. Under the same conditions, the Δ *vapA* mutant exhibited high sensitivity to all toxic analogues tested, indicating that these transporters are functional and reach the PM normally.

To directly assess PM localization, we examined the distribution of GFP-tagged versions of three representative non-polarized PM cargoes: the purine transporter UapA, the essential ATPase PmaA, and PalI. These constructs were introduced into the Δ *vapA* background via genetic crosses. As shown in Figure 9**B**, all three proteins localized correctly to the PM in the Δ vapA mutant, similarly to WT. Because UapA undergoes regulated endocytosis in response to ammonium addition 74, we also investigated whether this process is affected by VapA deletion. Figure 9**C** shows that UapA is internalized and sorted into vacuoles in Δ vapA cells just as in WT, indicating that its endocytosis proceeds normally.

In summary, our results demonstrate that while VapA is critical for the polarized, AP-2–dependent maintenance of apical cargoes, it is not required for the Golgi-independent trafficking or AP-2–independent endocytosis of non-polarized PM cargoes such as UapA, PmaA, and PalI. This distinction highlights the compartmentalized nature of endocytic pathways in *A. nidulans* and supports the idea that VapA specifically regulates endocytosis at the apical domain.

## DISCUSSION

### VapA is the sole ER-PM contact protein essential for proper *A. nidulans* growth

The physiological importance of VAPs in eukaryotes is underscored by studies showing that their genetic depletion in mammals is associated with several neurological disorders, including amyotrophic lateral sclerosis (ALS), Alzheimer’s disease (AD), and α -synucleinopathies such as Parkinson’s disease (PD) and multiple system atrophy (MSA) 75–77. Moreover, mammalian VAPs are hijacked by various viruses and intracellular bacteria to support their replication 13, 78. In plants, VAPs are also essential, playing key roles in lipid homeostasis, ER function, endocytosis, and interactions with fungi 79, 80.

In fungi, ER-PM contact site proteins have been predominantly studied in unicellular yeasts. Here, we genetically characterized all six predicted ER-PM contact site proteins in the model filamentous fungus *A. nidulans*, and found that only the single VAP/Scs2 homologue, VapA, is essential for proper growth and conidiospore formation. A similar growth defect has been observed upon deletion of the VAP/Scs2 orthologue in another filamentous fungus, the rice blast pathogen *M. oryzae* 35, where its loss also reduces virulence toward plant hosts. In contrast, deletion of VAP/Scs2 homologues in the yeasts *S. cerevisiae* and *S. pombe* causes only mild growth defects 8, 31, 46. This discrepancy likely reflects differences in growth strategies: unlike yeasts, filamentous fungi rely not only on the establishment but also the maintenance of cell polarity, which supports their development of long, asymmetric, hyphae rather than single cells 64, 68, 81.

To confirm VapA as an ER-resident protein, we functionally fused GFP to its N-terminus and showed colocalization with the ER marker Sec63, establishing it as an integral ER membrane protein. We further demonstrated that the C-terminal transmembrane (TM) domain is required for both ER anchoring and for rescuing the growth defect of the Δ *vapA* deletion mutant. Notably, as observed in *S. pombe* 34, full complementation of Δ *vapA* by GFP-VapA required high-level expression driven by a strong promoter, suggesting that VAP function is dosage-dependent in these fungi. Interestingly, C-terminal or internal GFP fusions of VapA in *A. nidulans* resulted in non-functional or unstable proteins, in contrast to *S. pombe*, where internally tagged Scs2 remains functional 34. Consistent with our findings, N-terminal GFP tagging has also been reported to produce functional VAP proteins in *S. cerevisiae* and *M. oryzae*.

### VapA is essential for localization of apical proteins involved in polarized growth via its role in specific lipid partitioning at the PM

Having established that VapA is a *bona fide* VAP homologue essential for proper growth, we next sought to understand the basis of the growth defect observed in the Δ *vapA* mutant. Since the genetic deletion of *vapA* had no apparent effect on unipolar germination or the initial stages of germling development, we hypothesized that the observed arrest in colony growth, including the failure to form mature mycelium and conidiospores, might stem from a defect in maintaining polarized growth. To test this, we examined the subcellular steady-state localization of apically localized protein cargoes involved in polarized growth, such as DnfA, DnfB, SynA, and ChsB. These proteins traffic to the hyphal tips via conventional, Golgi-dependent secretion, and they maintain their apical localization through constitutive endocytosis and recycling to the PM. Our results revealed that, although secretion to the PM was not significantly impaired in the absence of VapA, apical cargoes, including proteins related to lipid homeostasis (DnfA and DnfB, but also SynA), became depolarized, redistributing into the subapical segment of the PM, likely due to diffusion from the apical region. In line with this, we have provided evidence the VapA affects specific lipid partitioning at the PM, especially at the apical region. This evidence includes: a) altered filipin staining and increased sensitivity to itraconazole, indicating disrupted ergosterol partitioning at the PM, b) changes in the localization of pleckstrin homology (PH) domain markers, consistent with altered distribution of phospholipids such as PI4P in the Golgi and PI(4,5)P2 at the PM, c) mislocalization of the Sac1 phosphatase consistent with overaccumulation of PI4P and PI(4,5)P2, as all of the aforementioned changes are exhibited in the Δ vapA mutant. Previous studies across various systems have indeed established VapA as essential for proper lipid organization at the PM. Notice that we are aware that a direct effect of VapA on lipid homeostasis is missing form our work. This, however, would ideally necessitate highly challenging studies and particularly lipidomics of specific compartments of the cell (*i.e.*, apical versus subapical PM, or ER versus PM) that extend beyond the scope of this work. In addition, the use of pharmacological inhibitors, as those used in yeast and mammalian systems to inhibit lipid homeostasis-related kinases activity, has not established for *A. nidulans.*

### VapA is critical for AP-2 dependent endocytosis

An apical cargo depolarization phenotype, similar to the one observed in the *vapA* null mutant, had previously obtained in mutants defective in apical endocytosis, such as Δ *ap2* ${}^{\sigma }$ or *thiAp-slaB* 36, 37. Given that the AP-2 adaptor complex is a key regulator of cargo endocytosis at the hyphal tip, we hypothesized that VapA might be required for proper apical recruitment or activity of AP-2. Our data supported this idea: the absence of VapA phenocopied the effects of AP-2 dysfunction, affecting not only polarized growth and the distribution of ergosterol and phosphoinositides (PIs) in the PM, but also the apical localization of endocytic effectors such as SlaB and SagA. Thus, the simplest explanation for the cellular and developmental phenotypes observed in the Δ *vapA* mutant is that VapA, an ER-PM tethering protein, plays a critical role in facilitating AP-2-mediated endocytosis of lipid homeostasis related cargoes at the apical region. This function is likely essential for the polarized recycling of membrane proteins, and consequently, for maintaining long-term hyphal tip growth and colony development. How could VapA affect AP-2 function? Although VapA and AP-2 do not colocalize (Figure 4**F**), suggesting no physical interaction, our data indicate that VapA may influence AP-2 function indirectly through its critical role in specific lipid partitioning at the PM, particularly at the apical region. In line with this, previously published studies have shown that PI(4,5)P ${}_{2}$ directly regulates AP-2 recruitment by acting as a conformational switch 82, 83, and that phosphoinositide composition determines compartment-specific adaptor localization 84.

### VapA is not essential for transporter PM localization and endocytic turnover

Given the role of VapA in polarized cargo trafficking, we investigated whether it also affects the localization of non-polarized cargoes, such as the purine transporter UapA, the proton pump ATPase PmaA, and the alkaline pH sensor PalI. These proteins are not involved in apical cell expansion but are key for environmental sensing and adaptation, and PmaA is in fact necessary for growth via its pleiotropic action of pH homeostasis and nutrition. Notably, we previously showed that such non-polarized cargoes bypass the Golgi and are likely to reach the PM through alternative routes, possibly involving ER-PM contacts.

Importantly, through the present study, we provide direct evidence against this hypothesis. All three non-polarized cargoes tested trafficked normally to the PM in the Δ *vapA* mutant. Furthermore, toxic analogue growth assays indicated that other nutrient transporters remained functionally active in the absence of VapA. Supporting this, none of the ER-PM contact site mutants examined showed growth defects across a range of nutrients (Figure 1**C**), reinforcing that non-polarized nutrient transporters do not rely on ER-PM contacts for PM localization. In fact, we observed an increase in the steady-state PM levels of non-polarized cargoes in the Δ *vapA* mutant (see fluorescence intensity in Figure 8**B**). A similar increase in UapA translocation to the PM has also been reported in yeast Δ tether strains 85. These findings are consistent with recent work in *S. pombe* suggesting that ER-PM contacts can act as a physical barrier to vesicular secretion and protein exocytosis 34, 35.

Altogether, our results indicate that non-polarized trafficking to the PM in *A. nidulans* is independent of both the Golgi and ER-PM contact sites. VapA, although it had no co-localization with AP-2, may affect AP-2 function indirectly by shaping the lipid landscape of the PM. This altered lipid environment, especially perturbations in PI(4,5)P ${}_{2}$ distribution, could impair the formation or function of AP-2-dependent endocytic sites, thereby influencing the polarized trafficking of specific cargoes.

## MATERIALS AND METHODS

### Media, strains, growth conditions and transformation

Standard complete and minimal media for *A. nidulans* were used (FGSC, http://www.fgsc.net). Media and chemical reagents were obtained from Sigma-Aldrich (Life Science Chemilab SA, Hellas) or AppliChem (Bioline Scientific SA, Hellas). Glucose 1% (w/v) or fructose 0.1% (w/v) was used as carbon source. NH ${}_{4}^{+}$ (di-ammonium tartrate, (NH4) ${}_{2}$ C ${}_{4}$ H ${}_{4}$ O) or NaNO ${}_{3}$ were used as nitrogen sources at 10 mM. In growth tests with salt addition, KCl and NaH ${}_{2}$ PO ${}_{4}$ were added at a final concentration of 0.6 M and 0.5 M respectively. Nucleobases and toxic analogs were used at the following final concentrations: 5-fluorouracil (5-FU), 8-azaguanine (AZG), 5-fluorocytosine (5-FC) and oxypurinol (OX) at 100 μ M 82; uric acid at 0.5 mM. Itraconazole (ITZ) was used at a final concentration of 0.25 mg/L 11. Thiamine hydrochloride was used at a final concentration of 10–20 μ M as a repressor of the *thiA* ${}_{p}$ promoter in growth tests, microscopy or Western blot analysis 47. *A. nidulans* transformation was performed by generating protoplasts from germinating conidiospores using TNO2A7 83 or other *nkuA* DNA helicase deficient strains, that allow in-locus integrations of gene fusions via auxotrophic complementation. Integrations of gene fusions with fluorescent tags (GFP/mRFP/mCherry), promoter replacement fusions (*gpdA* ${}_{p}$ */thiA* ${}_{p}$ ) or deletion cassettes were selected using the *A. nidulans* marker para-aminobenzoic acid synthase (*pabaA*) or the *A. fumigatus* markers orotidine-5-phosphate-decarboxylase (AF*pyrG*, Afu2g0836), GTP-cyclohydrolase II (AF*riboB*, Afu1g13300) or a pyridoxine biosynthesis gene (AF*pyroA*, Afu5g08090), resulting in complementation of the relevant auxotrophies. To verify locus-specific integration of transformation cassettes, diagnostic PCRs were performed using genomic DNA extracted from transformants. Primer pairs were designed with one primer annealing outside of the homology region (either upstream or downstream of the targeted locus) and the second primer binding within the selectable marker gene. Only those transformants that yielded the expected PCR products for both 5 ${}^{\prime }$ and 3 ${}^{\prime }$ integration junctions were selected for further study. GFP-tagged versions of VapA were firstly introduced in AN4406/*vapA* genomic locus of TNO2A7 strain, but failed to complement WT VapA. To identify a functional GFP-tagged VapA, plasmids overexpressing GFP-VapA, VapA-GFP-TM and VapA- Δ TM-GFP under the *gpdA* ${}_{p}$ promoter, were introduced in Δ *vapA(AFpyrG) pantoB100*. GFP-VapA successfully complemented the null mutant (Figure 2**B**). To that end, the plasmid carrying *gpdA* ${}_{p}$ -GFP-VapA was used for transformation of Δ 7 ( Δ *furD::riboB* Δ *furA::riboB* Δ *fcyB::argB* Δ *azgA* Δ *uapA* Δ *uapC::AfpyrG* Δ *cntA::riboB pabaA1 pantoB100*), based on complementation of the pantothenic acid auxotrophy *pantoB100* 73. Transformants were verified by PCR and growth test analysis. Combinations of mutations and fluorescent epitope-tagged strains were generated by standard genetic crossing and progeny analysis. *E. coli* strains used were DH5a. *A. nidulans* strains used are listed in Supplementary Table S1-Strain List. There is also a list containing annotations for genes referenced in the present work Supplementary Table S2-Annotations.

### Nucleic acid manipulations and plasmid constructions

Genomic DNA extraction was performed as described in FGSC (http://www.fgsc.net). Plasmid preparation and DNA gel extraction were performed using the Nucleospin Plasmid and the Nucleospin Extract II kits (Macherey-Nagel, Lab Supplies Scientific SA, Hellas). Restriction enzymes were from NEB (New England Biolabs, Bioline Scientific SA, Hellas). DNA sequences were determined by Eurofins-Genomics (Vienna, Austria). Conventional PCRs reactions were performed with KAPA Taq DNA polymerase (Kapa Biosystems, Lab Supplies Scientific). High-fidelity amplification of products and site-directed mutagenesis were performed with Kapa HiFi polymerase (Kapa Biosystems, Lab Supplies Scientific). Gene cassettes were generated by sequential cloning of the relevant fragments in the pGEM-T plasmid (Promega), which served as template to PCR-amplify the relevant linear cassettes. The genomic sequences for AN4406/*vapA,* AN9149/*tcbA*, AN5624/*tcbB*, AN2477/*istA*, AN7165/*istB* were retrieved from FungiDB (https://fungidb.org/fungidb/app). For AN4406/*vapA* deletion, several constructs were generated using *pabaA*, AF*pyroA* and AF*pyrG* genes. To construct VapA-GFP-TM and VapA- Δ TM-GFP, SOSUI-transmembrane helix prediction tool (https://harrier.nagahama-i-bio.ac.jp/sosui/mobile/) was used, to identify a 22 amino acids transmembrane helix on the C-terminus of VapA (258-280aa, AGVPVRIVAGLCLLSFLIAYFFF). For the overexpression of GFP-tagged versions of *vapA,* a modified pGEM-T-easy vector was used, carrying a version of the *gpdA* promoter, the *trpC* 30 termination region, and the *pantoB* selection marker 84. Oligonucleotides used for cloning are listed in Supplementary Table S3-Primer List.

### Phylogenetic tree

Protein sequences were retrieved from the UniProt database (https://www.uniprot.org/) for a representative selection of model organisms. All sequences were manually curated to ensure orthology and high annotation quality. Multiple sequence alignment was conducted using the MAFFT online server 86 at https://mafft.cbrc.jp/alignment/server/, applying the E-INS-i algorithm. Alignment settings included two iterative refinement cycles, and the “Try to align gappy regions anyway” option was selected to preserve informative gaps. The final alignment comprised 23 sequences with 557 amino acid positions. Phylogenetic tree was constructed using the IQ-TREE web server v1.6.12 87, 88 with integrated ModelFinder 89 for automated substitution model selection. The best-fitting model according to the Bayesian Information Criterion (BIC) was LG+I+G4, which includes a general amino acid replacement matrix (LG), a proportion of invariable sites (I), and gamma-distributed rate heterogeneity across four discrete categories (G4). Maximum likelihood (ML) tree inference was performed under this model, and ultrafast bootstrap approximation (UFBoot2) was applied with 1,000 replicates to assess branch support 90, 91. The resulting unrooted ML tree was rooted using the plant clade as the outgroup. The tree was visualized and annotated using FigTree v1.4.4 (http://tree.bio.ed.ac.uk/software/figtree/). Supplementary Table S4 compiles the uniport IDs used to generate the tree. Note that for AN4406/VapA, the sequence was retrieved from FungiDB (https://fungidb.org/fungidb/app), as there were missing residues on the C-tail at the Uniprot sequence (Q5B4X4).

### Protein extraction and Western blots

Protein extraction and western blotting was performed as previously described by 57, 70. For strains shown in Supplementary Figure S1 and Supplementary Figure S3, dry mycelia from cultures grown in minimal liquid cultures supplemented with 10 mM NH ${}_{4}^{+}$ , and the required auxotrophies were used (culture conditions: 25 ${}^{\circ }$ C for 16 h). For *thiA* ${}_{p}$ -*tcbB,* thiamine hydrochloride was added at a final concentration of 20 μ M. Total proteins (50 μ g, estimated by Bradford assays) were separated in a 10% (w/v) poly acrylamide gel and then transferred on PVDF membranes (GE Health care Life Sciences, Amersham). Immunodetection was performed with an anti-FLAG antibody (Agrisera, AAS15 3037) for *thiA* ${}_{p}$ -FLAG-*tcbB* or an anti-GFP antibody (11814460001, Roche Diagnostics) for *gpdA* ${}_{p}$ -GFP-VapA constructs. Then an HRP-linked anti body (7076, Cell Signaling Technology Inc.) was used in both cases. Colloidal Coomassie G-250 Staining for Proteins (CBB) was employed as described in Dyballa and Metzger, 2009, to ensure equal loading. For *thiA* ${}_{p}$ -FLAG-*tcbB* Western blot, an anti-actin monoclonal (C4) antibody (SKU0869100-CF, MP Biomedicals, Europe), was also employed. Blots were developed using the Lumi Sensor Chemiluminescent HRP Substrate kit (Genscript, United States) and SuperRX Fuji medical X-Ray films (Fuji FILM, Europe).

### Fluorescence microscopy

Conidiospores were incubated overnight in glass bottom 35 mm μ -dishes (ibidi, Lab Supplies Scientific SA, Hellas) in liquid minimal media containing 1% (w/v) glucose, supplemented with NH ${}_{4}^{+}$ , and the required auxotrophies, for 16–22 h at 25 ${}^{\circ }$ C. For following the subcellular trafficking and localization of DnfA, DnfB, ChsB, UapA, PmaA, PalI, these cargoes were expressed under their native promoter. The *uapA* promoter can be tightly repressed in the presence of 10 mM ammonium tartrate supplied as a nitrogen source in the growth medium, and derepressed in the presence of nitrate. mCherry-SynA on wtand Δ *ap2* $\${}^{\sigma }\$$ strain was expressed under its native promoter. GFP-SynA was expressed under the regulatable *alcA* promoter in wtand Δ *vapA* strains. *AlcA* ${}_{p}$ is repressed on 1% w/v glucose and derepressed upon shift to 0.1% w/v fructose for 4–5 h. For following the subcellular localization of *de novo* GFP-OshB, the transcription was induced upon shift to 0.1% w/v fructose and 0.4% v/v ethanol containing media for 4 h. The exact same conditions were used to track the localization of DnfA-GFP controlled by *alcA* ${}_{p}$ promoter, and images were obtained at 1.5, 2 and 4 hours upon induction respectively. Selected proteins (*thiA* ${}_{p}$ *-myoA, thiA* ${}_{p}$ *-slaB*) expressed under the *thiA* promoter, were transcriptionally repressed in the presence of 10 μ M thiamine in the growth media. SynaptoRed C2 (Equivalent to FM4-64) (Biotium) staining took 5–8 min on ice at a final concentration of 4 μ M, and observed at 5 min and 25 min post-washes. CellTracker™ Blue CMAC (InvitrogenTM) was used according to 44. FilipinIII (Cayman Chemical) staining took place 5 min, room temperature at 1 μ g/ml final concentration 36. Calcofluor white was used at 0.001% w/v final concentration, for 5 min, room temperature (RT) 36. Images from widefield microscopy were obtained using an inverted Zeiss Axio Observer Z1 equipped with the white light pE-400 Illumination System (https://www.coolled.com/products/pe-400) and a Hamamatsu ORCA-Flash 4 camera. All widefield z-stack images were deconvolved with Huygens Essential version 23.10 (Scientific Volume Imaging, The Netherlands, http://svi.nl). Technical replicates correspond to different hyphal cells observed within each sample, while biological replicates refer to different samples. Each experiment has been conducted at least two times.

### Image processing and statistical analysis

Images of fungal colonies from growth assays were processed for comparative presentation using Adobe Photoshop CS4 Extended (version 11.0.2). Individual colonies representing different *A. nidulans* strains were isolated from Petri dishes photographs, using the “layer via Copy” tool, allowing rearrangement of colonies from different plates into a single composite figure. (Figures 1**C**, 2**A**, 3**A**, 5**A**, 6**C**, 9**A**, Supplementary Figure S1, Supplementary Figure S2). Adjustments to brightness and contrast were applied to improve visibility across samples. All image processing steps were limited to figure preparation and did not affect the experimental analysis. Each growth test has been conducted at least two times. Microscopy images were processed and analyzed using Fiji/ImageJ 91. Maximum intensity projections, contrast adjustments, region-of-interest (ROI) selection, color channel merging, and scale bar insertion were performed to enhance image clarity for figure presentation. These manipulations were applied uniformly, after any fluorescence intensity measurement and did not alter the underlying fluorescence signals used for quantification. Additional image annotation and formatting were performed using Adobe Photoshop CS4 Extended (version 11.0.2). To quantify cER fluorescence labeled with Sec63-mCherry (Figure 4**A**), the polygon selection tool in Fiji was used to define a ROI approximately 10–12 μ m from the hyphal tip (ROI_total). A second, smaller ROI within this region was drawn to measure the cytoplasmic signal (ROI_intracellular). Fluorescence intensities were normalized to area, and cER fluorescence per μ m was calculated by subtracting the intracellular signal from the total:

Cortical ER Fluorescence/Area = (Total IntDen/Area) – (Intracellular IntDen/Area). The resulting values were compared between wild-type (WT) and Δ *vapA* strains using an unpaired *t*-test. The mean ± SEM for WT was 90.63 ± 12.64 (N = 13), and for Δ vapA was 107.7 ± 15.29 (N = 17), with t = 0.8263, degrees of freedom (df) = 28, and p = 0.4156. To assess ER– PM contact sites marked by TcbA-GFP (Figure 4**A**), a segmented line approximately 8–12 μ m in length was drawn along the PM at the hyphal tip. Fluorescence intensity profiles were generated using Fiji/ImageJ, and regions with intensity values greater than 1000 units were considered TcbA-enriched. The length of TcbA-positive membrane was divided by the total measured membrane length to yield a ratio per cell. WT cells showed a mean ratio of 0.6575 ± 0.07126 (N = 17), and Δ *vapA* cells showed 0.5415 ± 0.06840 (N = 20), with t = 1.171, df = 35, and p = 0.2495. For SedV and PH ${}^{\mathrm{OSBP}}$ (Figure 4**A**), fluorescence intensity was measured using a polygon ROI starting approximately 8 μ m from the hyphal tip, with each value corresponding to a single hypha (technical replicate). For SedV, WT had a mean intensity of 875.4 ± 65.84 (N = 24) and Δ *vapA* had 706.7 ± 47.21 (N = 28), yielding t = 2.123, df = 50, and p = 0.0387. For PH ${}^{\mathrm{OSBP}}$ , Welch’s *t*-test was used due to unequal variances: WT had a mean of 1835 ± 409.7 (N = 20) and Δ *vapA* had 4207 ± 840.0 (N = 20), with t = 2.538, df = 27, and p = 0.0172. For nuclei (H1-mRFP) and peroxisomes (mRFP-AKL), the number of fluorescent structures was counted per 15 μ m hyphal segment. The average values of WT and Δ *vapA* strains were compared using unpaired t-tests. For nuclei (H1), WT had a mean ± SEM = 1.647 ± 0.1906 ( n=17 ) and Δ *vapA* = 1.643 ± 0.1991 (n = 14; t=0.01516 , df = 29, p=0.9880 ), indicating no significant difference. For peroxisomes (AKL), WT = 6.100 ± 0.5667 ( n=10 ) and Δ *vapA* = 4.917 ± 0.5288 (n = 12; t=1.523 , df = 20, p=0.1433 ), which was also not significant (p > 0.05). All statistical analyses for the Sec63, TcbA, SedV, and PH ${}^{\mathrm{OSBP}}$ experiments were performed using GraphPad Prism3 (GraphPad Software, San Diego, CA, USA).

Full datasets and statistical outputs for these comparisons are available in Data_Fig4. For quantification of apical fluorescence gradients (Figures 5**C**, 7 and Supplementary Figure S4), a line scan analysis was performed. A straight line was drawn from the hyphal tip extending 5 μ m into the cell interior. Fluorescence intensity along this line was measured using the line profile tool in Fiji. This procedure was repeated for each hypha, and the average intensity at each position along the line was calculated for each strain. For region-specific analysis, intensity values were binned into 1 μ m intervals (*i.e.*, 0–1 μ m, 1–2 μ m, 2–3 μ m, 3–4 μ m, and 4–5 μ m). Within each bin, fluorescence values for each strain were pooled, and pairwise comparisons were conducted between WT and Δ *vapA*, as well as WT and Δ *ap2* $\${}^{\sigma }\$$ . Statistical outputs are included in Data_Fig5 and Data_Fig7. A similar method was applied in **Figures 4B, 4C, 5E, 6A, and 7** to quantify cortical fluorescence intensity surrounding the PM at the hyphal tip. In these experiments, segmented lines were drawn along the PM covering distances of 0–10 μ m or 0–12 μ m, depending on the figure. Fluorescence values along these lines were binned by 1 μ m and analyzed similarly to the apical gradient measurements. Statistical analyses for these figures are provided in Data_Fig4, Data_Fig5, Data_Fig6, and Data_Fig7. For FM4-64 internalization experiments (Figure 6D), the polygon selection tool in Fiji was used to define a ROI approximately 10 μ m from the hyphal tip (ROI_total). A second, smaller ROI within this region was drawn to measure the cytoplasmic signal (ROI_intracellular). Fluorescence intensities were normalized to area, and PM fluorescence per μ m was calculated by subtracting the intracellular signal from the total: PM Fluorescence/Area = (Total IntDen/Area) – (Intracellular IntDen/Area). For our analysis the Intracellular IntDen/Area corresponds to the Cytosolic fluorescence per μ m. The resulting values were grouped for the two conditions used: 5 and 25 min FM4-64 internalization respectively. Unpaired *t*-test to compare the PM and cytosolic fluorescence intensity of wild-type (WT) and Δ *vapA* was employed. Notably, only the comparison of the cytosolic fluorescence intensity at 25 min, proved to be statistically significant (p < 0.05, p = 0.0308, t = 2.201 df = 75, N ${}_{\mathrm{WT}}$ = 38, N ${}_{\Delta \mathrm{vapA}}=39$ ).

Statistical analyses for the apical fluorescence gradient and cortical fluorescence intensity measurement, were performed using a custom Python script (Python v3.10) utilizing pandas, NumPy, and SciPy.Stats libraries 92–94, 97–99. A 95% confidence interval was applied for all tests. Prior to hypothesis testing, data normality was assessed using the Shapiro–Wilk test, and homogeneity of variances was evaluated using Levene’s test. If both assumptions were met, an unpaired *t*-test was used; if variances were unequal, Welch’s *t*-test was applied; and if normality was violated in either group, the non-parametric Mann–Whitney U test was employed. All fluorescence intensity plots were generated using GraphPad Prism3. For co-localization analysis, Pearson’s correlation coefficient (PCC) was calculated using the BIOP JACoP plugin in Fiji 95, 100. PCC values were computed from maximum intensity projections of deconvolved z-stacks, using manually defined ROIs that encompassed each hypha. Statistical analysis and visualization of PCC values were performed using GraphPad Prism, and a one-sample *t*-test with a 95% confidence interval was used to determine significance.

### Dry mycelia biomass measurement

To assess fungal biomass over time, we measured the dry weight of mycelia from wild-type (wt) and Δ *vapA* strains on liquid culture. For each strain, 900 μ l of a freshly prepared spore suspension (6.5 × 10 ${}^{6}$ spores/ml, quantified using a Neubauer) was inoculated into 25 ml of liquid minimal medium containing 1% (w/v) glucose, 10 mM NH ${}_{4}^{+}$ as the nitrogen source, and supplemented with the appropriate auxotrophies. Cultures were incubated at 25 ${}^{\circ }$ C with shaking at 140 rpm for 16, 24, and 48hours. For each time point, three biological replicates (*i.e.*, independent 25ml cultures) were analyzed per strain. At each time point, mycelia were harvested by filtration through blutex filters and dried at 60 ${}^{\circ }$ C for a minimum of 24 hours. Dried mycelia were then weighed, and the recorded values were used for statistical analysis. Unpaired two-tailed t-tests were performed using GraphPad Prism, to assess differences in biomass between strains. At 16 h, no statistically significant difference was observed between wtand Δ vapA (p = 0.2063, t = 1.507, df = 4). At 24 h, a significant reduction in biomass was detected in the Δ vapA strain (p = 0.04, t = 2.998, df = 4). At, 48 h again there was significant reduction in Δ vapA strain (p = 0.0072, t = 5.054, df = 4).

## DATA AVAILABILITY STATEMENT

Strains and plasmids are available upon request. The authors affirm that all data necessary for confirming the conclusions of the article are present within the article, figures, and tables. Raw images or data are available upon request.

## SUPPLEMENTAL MATERIAL

All supplemental data for this article are available online at http://microbialcell.com/researcharticles/2026a-georgiou-microbial-cell/.



## CONFLICT OF INTEREST

The authors declare no conflict of interest.

## ABBREVIATIONS

PM – plasma membrane

ER – endoplasmic reticulum

wt – wild-type

cER – cortical ER

TM – transmembrane

PI(4,5)P_2_-Phosphatidylinositol (4,5) – bisphosphate

PI4P – Phosphatidylinositol 4-phosphate

MCS – membrane contact sites
