# Supplementary material for: VapA/Scs2 sustains polarized growth in Aspergillus nidulans by maintaining AP-2-mediated apical endocytosis
Source: Microb Cell. 2026 Feb 4;13:63–85. doi: 10.15698/mic2026.02.868 (PMC12925633; doi:10.15698/mic2026.02.868)

Supplementary information

Supplementary Figures

Supplementary figure S1: Extended growth test, part of which is presented in **Figure1C**. Strains carrying single total deletions of genes encoding VapA/VAP, tricalbins or Ist2 proteins (Δ*vapA*, Δ*tcbA*, Δ*istA* and Δ*istB*). Growth of doubly knockout mutant *ΔistA/ΔistB* is also shown. A standard wild-type strain (wt) and a strain carrying multiple deletions in genes encoding purine/pyrimidine-related transporters are used as controls (Δ*furD* Δ*furA* Δ*fcyB* Δ*uapA* Δ*uapC* Δ*azgA* Δ*cntA*, named Δ7). Strains are grown on minimal media with selected nitrogen sources (ammonium/NH_4^+ or nitrate/NO_3^- or uric acid/UA), at pH=6.8, at 25 °C, 37°C, 42 °C. **B.** Western blot analysis of TcbB (AN5624) using the anti- FLAG antibody. In the absence of thiamine (−thi) from the growth medium, TcbB is expressed, while upon addition of thiamine (+thi) at the onset of conidiospore germination (ab initio repression), the expression of these proteins is tightly repressed. Equal loading and protein steady-state levels are normalized against the amount of actin, detected with an anti- actin antibody. **C.** Bar plot showing the dry weight (mg) of mycelia harvested from wild-type (wt) and Δ*vapA* strains grown in liquid minimal medium at 25 °C for 16, 24, and 48 hours. For each condition, three biological replicates (independent 25 ml cultures) were analyzed. Mycelia were filtered, dried at 60 °C for ≥24 h, and weighed. Statistical comparisons were performed using an unpaired two-tailed t-test (see materials and methods section). $p < 0.05$ was considered significant; Notably, there is significant reduction on Δ*vapA* biomass at 24h and 48h compared with the wt, while there is no significant difference at 16h of liquid culture.

Supplementary figure S2: Growth phenotypes of strains carrying single total deletions of genes encoding VapA/VAP, tricalbins or Ist2 proteins (Δ*vapA*, Δ*tcbA*, Δ*istA* and Δ*istB*). Growth of doubly knockout mutant *ΔistA/ΔistB* is also shown. A standard wild-type strain (wt) and a strain carrying multiple deletions in genes encoding purine/pyrimidine-related transporters are used as controls (Δ*furD* Δ*furA* Δ*fcyB* Δ*uapA* Δ*uapC* Δ*azgA* Δ*cntA*, named Δ7). Strains are grown on minimal media with selected nitrogen sources (ammonium/NH_4^+ or nitrate/NO_3^- or uric acid/UA), at pH=5.5 and 8, at 25 °C, 37°C, 42 °C.

Supplementary figure S3: Western blot analysis of GFP-VapA, VapA-GFP-TM, VapA-ΔTM-GFP, under the *gpdA* promoter in Δ*vapA* background using the anti- GFP antibody. Notice there is high expression of the VapA-GFP construct, estimated at 63 kDa, while VapA-GFP-TM is barely detected and VapA-ΔTM-GFP exhibits increased degradation. Equal loading was achieved using Coomassie staining.

Supplementary figure S4: mCherry-SynA localization in wt and Δ*ap2^σ^*.

Maximal intensity projections of deconvolved snap shots showing the localization of mCH-SynA in wt and Δ*ap2*$^{\sigma}$ background. Notice that SynA is unaffected on Δ*ap2*$^{\sigma}$. Line plot showing SynA fluorescence intensity along the hyphal tip in wild-type (WT, red) and Δ*ap2*$^{\sigma}$ (green) strains. The x-axis represents the distance from the hyphal tip (μm), and the y-axis represents fluorescence intensity.

Supplementary figure S5. Comparison of DnfA and ChsB localization in Δ*vapA*, Δ*ap2*$^{\sigma}$, *thiAp-myoA* and *thiAp-slaB*.
Notice that upon repression of endocytosis factors, MyoA and SlaB, DnfA and ChsB have totally lost their apical localization and mark homogeneously the PM. In Δ*ap2*$^{\sigma}$, only DnfA exhibits severe endocytosis defect, while ChsB remains apical yet labels intracellular loci. A similar phenotype for ChsB, was observed also in Δ*vapA*. Scale bars: 5 μm

Supplementary Tables

Supplementary Table S2: Annotations

FungiDB ID	Systematic/Protein name	Cellular process
**AN4406**	VapA/ Scs2/22	ER-PM tethering
**AN9149**	TcbA	ER-PM tethering
**AN5624**	TcbB	ER-PM tethering
**AN7165**	Ist2B	ER-PM tethering
**AN2477**	Ist2A	ER-PM tethering
**AN0722**	Ap2σ	Endocytosis
**AN2756**	SlaB	Endocytosis
**AN1023**	SagA	Endocytosis
**AN12237**	AbpA	Endocytosis
**AN1558**	MyoA	Endocytosis
**AN7682**	Ap1σ/Aps1	Late Golgi sorting
**AN9526**	SedV/Sed5	Qa-SNARE
**AN8769**	SynA/Snc1/2	R-SNARE
**AN8672**	DnfA	apical cargo
**AN6112**	DnfB	apical cargo
**AN2523**	ChsB/Chs3	apical cargo
**AN4859**	PmaA	non-polar cargo
**AN4853**	PalI	non-polar cargo
**AN6932**	UapA	non-polar cargo
**AN3424**	OshB/Osh3	Oxysterol-binding
**AN3841**	Sac1	PI catabolism
**AN1901**	Erg11A	Ergosterol biosynthesis
**AN8283**	Erg11B	Ergosterol biosynthesis
**AN2684**	Erg4A	Ergosterol biosynthesis
**AN10648**	Erg4B	Ergosterol biosynthesis
**AN0913**	PisA	PI biosynthesis
**AN0640**	BasA	Sphingolipids biosynthesis

Supplementary Table S3: Primers

Plasmids	Oligonucleotides	5'3' Sequence
pGEM *ΔvapA/ΔAN4406::AFpyrG*	5 up AN4406 ApaI F	CACAGGGCCCCGACCTGCTTTCTCATCGCTGTC
	5 up AN4406 SpeI R	CTGACTAGTTCCAAAGACACGGCGGTACCT
	3 down An4406 SpeI F	GCGCACTAGTCCCTTGAGCCGTCTGTCTCTGATT
	3 down AN4406 NotI R	GCGCGCGGCCGCGGACAAAACTTCATACCCTCCACCG
	AFpyrG SpeI F	CGCGACTAGTGCCTCAAACAATGCTCTTCACCCTC
	AFpyrG SpeI R	CGGACTAGTCTGTCTGACAGGAGGCACTGATGCG
pGEM *ΔvapA/ΔAN4406::AFpyroA*	5 up AN4406 ApaI F	CACAGGGCCCCGACCTGCTTTCTCATCGCTGTC
	5 up AN4406 SpeI R	CTGACTAGTTCCAAAGACACGGCGGTACCT
	3 down An4406 SpeI F	GCGCACTAGTCCCTTGAGCCGTCTGTCTCTGATT
	3 down AN4406 NotI R	GCGCGCGGCCGCGGACAAAACTTCATACCCTCCACCG
	AFpyroA SpeI F	CGCGACTAGTGGACATCAGATGCTGGATTAC
	AFpyroA SpeI R	CGCGACTAGTGCGAGTGTCTACATAATGAAGG
pGEM *ΔvapA/ΔAN4406::pabaA1*	5 up AN4406 ApaI F	CACAGGGCCCCGACCTGCTTTCTCATCGCTGTC
	5 up AN4406 SpeI R	CTGACTAGTTCCAAAGACACGGCGGTACCT
	3 down An4406 SpeI F	GCGCACTAGTCCCTTGAGCCGTCTGTCTCTGATT
	3 down AN4406 NotI R	GCGCGCGGCCGCGGACAAAACTTCATACCCTCCACCG
	paba-XbaI-F	GGCTCTAGACCTGTCAAATTGGCAGATAGG
	paba-XbaI-R	GGCTCTAGACCGACCCATCTATCGCG
pGEM *AN4406/vapA-GFP-TM::AFpyrG*	orf4406 ATG ApaI F	GTCAGGGCCCATGTCCATCCTCCTCGACC
	orf4406 no TM SpeI R	GCTCACTAGTCTCAACAGACTGAACCTGCTGC
	GFP SpeI F2	GCCACTAGTATGGTGAGCAAGGGCGAG
	GFP ns TM 4406 SpeI R	ACTGACTAGTTTAGAAAAAGAAATACGCAATCAAGAAACT CAGCAGGCATAAACCTGCCACAATCCTTACTGGCACACCT GCCTTGTACAGCTCGTCCATGCC
	3 down An4406 SpeI F	GCGCACTAGTCCCTTGAGCCGTCTGTCTCTGATT
	3 down AN4406 NotI R	GCGCGCGGCCGCGGACAAAACTTCATACCCTCCACCG
	AFpyrG SpeI F	CGCGACTAGTGCCTCAAACAATGCTCTTCACCCTC
	AFpyrG XbaI R	CGGTCTAGACTGTCTGAGAGGAGGCACTGATGCG
pGEM *AN4406/vapAΔTM-GFP::AFpyrG*	orf4406 ATG ApaI F	GTCAGGGCCCATGTCCATCCTCCTCGACC
	orf4406 no TM SpeI R	GCTCACTAGTCTCAACAGACTGAACCTGCTGC

	GFP SpeI F2	GCCACTAGTATGGTGAGCAAGGGCGAG
	GFP stop SpeI R	GCTGACTAGTTTACTTGTACAGCTCGTCCATGCC
	3 down An4406 SpeI F	GCGCACTAGTCCCTTGAGCCGTCTGTCTCTGATT
	3 down AN4406 NotI R	GCGCGCGGCCGCGGACAAAACTTCATACCCTCCACCG
	AFpyrG SpeI F	CGCGACTAGTGCCTCAAACAATGCTCTTCACCCTC
	AFpyrG XbaI R	CGGTCTAGACTGTCTGAGAGGAGGCACTGATGCG
pGEM *AN4406/vapA-GFP::AFpyrG*	orf4406 ATG ApaI F	GTCAGGGCCCATGTCCATCCTCCTCGACC
	orf4406 ns SpeI R	GCTGACTAGTGAAAAAGAAATACGCAATCAAGAAACTCAG c
	GFP SpeI F2	GCCACTAGTATGGTGAGCAAGGGCGAG
	GFP stop SpeI R	GCTGACTAGTTTACTTGTACAGCTCGTCCATGCC
	AFpyrG SpeI F	CGCGACTAGTGCCTCAAACAATGCTCTTCACCCTC
	AFpyrG XbaI R	CGGTCTAGACTGTCTGAGAGGAGGCACTGATGCG
	3 down An4406 SpeI F	GCGCACTAGTCCCTTGAGCCGTCTGTCTCTGATT
	3 down AN4406 NotI R	GCGCGCGGCCGCGGACAAAACTTCATACCCTCCACCG
pGEM *GFP-(GA)X5-AN4406/vapA::AFpyrG*	5 up AN4406 ApaI F	CACAGGGCCCCGACCTGCTTTCTCATCGCTGTC
	5 up AN4406 SpeI R	CTGACTAGTTCCAAAGACACGGCGGTACCT
	GFP SpeI F2	GCCACTAGTATGGTGAGCAAGGGCGAG
	GFPnsGA SpeI R	GCGACTAGTGGCACCGGCTCCAGCGCCTGCACCAGCTCCct tgtacagctcgtccatgcc
	AFpyrG SpeI F	CGCGACTAGTGCCTCAAACAATGCTCTTCACCCTC
	AFpyrG XbaI R	CGGTCTAGACTGTCTGAGAGGAGGCACTGATGCG
	AN4406 ORF ATG SpeI F	TGTGACTAGTATGTCCATCCTCCTCGACC
	AN4406 ORF stop NotI R	GACTGCGGCCGCTTAGAAAAAGAAATACGCAATCAAGAA ACTCAG
pGEM *gpdAp-GFP-(GA)x5-AN4406/vapA-trpC-pantoB*	GFP SpeI F2	GCCACTAGTATGGTGAGCAAGGGCGAG
	GFPnsGA SpeI R	GCGACTAGTGGCACCGGCTCCAGCGCCTGCACCAGCTCCct tgtacagctcgtccatgcc
	AN4406 ORF ATG SpeI F	TGTGACTAGTATGTCCATCCTCCTCGACC
	AN4406 ORF stop NotI R	GACTGCGGCCGCTTAGAAAAAGAAATACGCAATCAAGAA ACTCAG
pGEM *gpdAp-mCherry-(GA)x2-AN4406/vapA-trpC-pantoB*	mCherry XbaI F	GCGCTCTAGAATGGTGAGCAAGGGCGAGGAG
	mCherry NS GA XbaIR	GCGCTCTAGATGCTCCCTTGTACAGCTCGTCCATGCC
	AN4406 ORF ATG SpeI F	TGTGACTAGTATGTCCATCCTCCTCGACC
	AN4406 ORF stop NotI R	GACTGCGGCCGCTTAGAAAAAGAAATACGCAATCAAGAA ACTCAG
pGEM *gpdAp-AN4406*	AN4406 ORF ATG SpeI F	TGTGACTAGTATGTCCATCCTCCTCGACC

	orf4406 no TM SpeI R	GCTCACTAGTCTCAACAGACTGAACCTGCTGC
	GFP SpeI F2	GCCACTAGTATGGTGAGCAAGGGCGAG
	AN4406 ORF stop NotI R	GACTGCGGCCGCTTAGAAAAAGAAATACGCAATCAAGAAACTCAG
**pGEM-gpdA-AN4406/vapAΔ TM-GFP-trpC-pantoB**	AN4406 ORF ATG SpeI F	TGTGACTAGTATGTCCATCCTCCTCGACC
	orf4406 no TM SpeI R	GCTCACTAGTCTCAACAGACTGAACCTGCTGC
	GFP SpeI F2	GCCACTAGTATGGTGAGCAAGGGCGAG
	GFP stop NotI R	CGCGCGGCCGCTTACTTGTACAGCTCGTCCATGC
**pGEM *ΔtcbA/ΔAN9149::AFpyrG***	5' up AN9149 ApaI F	GTCAGGGCCCCGGAGCATGTGTGGCGCATG
	5' up AN9149 SpeI R	ACTGACTAGTGCCAAGACGCTCGTAACGTTTTCG
	3' down SpeI AN9149 F	TCTGACTAGTTGTTAGTCTACCGAAGACCCCTCCC
	3' down AN9149 NotI R	TCAGCGGCCGCCGCAGGCCAGATACAAGACAGATCG
	AFpyrG SpeI F	CGCGACTAGTGCCTCAAACAATGCTCTTCACCCTC
	AFpyrG SpeI R	CGGACTAGTCTGTCTGACAGGAGGCACTGATGCG
**pGEM *AN9149/tcbA-(GA)X5-GFP::AFpyrG***	3 ORF ns AN9145 ApaI F	CCGGGGCCCCGTACGTCATGCGCTCTCGCC
	3 ORF ns AN9149 SpeI R	GCGCACTAGTCGCCCCGCTGACGCTGC
	GAGFP XbaI F	GCGTCTAGAGGAGCTGGTGCAGGCGCTGGA
	GAGFP SpeI R	CGCGACTAGTTTATTTGTATAGTTCATCCATGCCATG
	AFpyrG SpeI F	CGCGACTAGTGCCTCAAACAATGCTCTTCACCCTC
	AFpyrG XbaI R	CGGTCTAGACTGTCTGAGAGGAGGCACTGATGCG
	3' down AN9149 SpeI F	TCTGACTAGTTGTTAGTCTACCGAAGACCCCTCCC
	3' down AN9149 NotI R	TCAGCGGCCGCCGCAGGCCAGATACAAGACAGATCG
**pGEM *Δist2a/ΔAN2477::AFriboB***	5' up AN2477 SphI F	GTCAGCATGCGGGAGTTCGGAGGCTATACGC
	5' up AN2477 SpeI R	GTCACTAGTCGTCATCCGCCCAAGCTGGA
	3' down AN2477 SpeI F	CTGACTAGTGCTCCGTACAACTAGCCAGAACC
	3' down AN2477 NotI R	ACAGCGGCCGCGGAAGAGGAGGGAAAGTTACGGTGAC
	AFriboB SpeI F	CGCGACTAGTCCCGGGCTGCAGGAATTCG
	AFriboB SpeI R	CGCGACTAGTCCCGGGCTGCAGGAATTCGA
**pGEM *Δist2b/ΔAN7165::AFpyrG***	5' up AN7165 ApaI F	GCGCGGGCCCGCATTGGCGTCAGGTCGAGCAGAG
	5' up An7165 XbaI R	GCGCTCTAGAGCGACTAATTCATCCAGGCAC
	3' down AN 7165 XbaI F	GCGCTCTAGAGTGCACCGAGCATGAGTTG

	3' down NotI R	CCGGGCGGCCGCCGCATTCTGACACTGAATGACGTGG
	AFpyrG SpeI F	CGCGACTAGTGCCTCAAACAATGCTCTTCACCCTC
	AFpyrG SpeI R	CGGACTAGTCTGTCTGACAGGAGGCACTGATGCG
**pGEM *thiAp-FLAG-tcbB/AN5624*::AFriboB**	new 5' up AN5624 ApaI F	GCCCGGGCCCGCTGTGTCAATGGATTGCACCG
	new 5 up AN5624 SpeI R	GGCCACTAGTCGGTGATGTCACCACGATCTCC
	5' orf AN5624 SpeI F	GTGACTAGTATGCCTGCTCACCCAAAGGG
	5' ORF AN5624 NotI R	GTCAGCGGCCGCATCACACCAGGTGAGGACGC
	AFriboB SpeI F	CGCGACTAGTCCCGGGCTGCAGGAATTCG
	AFriboB XbaI R	CGCGTCTAGACCCGGGCTGCAGGAATTCGATAAG
	thiAp XbaI F	CGCGTCTAGACGACCTGGCACCTACAGAAGAATC
	thiA FLAG XbaI R	CGCGTCTAGACTTGTCATCGTCGTCCTTGTAGTCCATGTTG ACTCAGTTCAATGGTTCGACTATAG
**pGEM *thiAp-sac1/AN3841*::AFriboB**	AN3841 5 ApaI F	CGCGGGGCCCCGAGGTGCTGTTCGCATGTTTCG
	AN3841 5 SpeI R	CGCGACTAGTTGCGGCGACGCCCACTTGTGCAG
	AN3841 ORF SpeI F	CGCGACTAGTATGGTCACTTCTATCCTCCCTTTTCG
	AN3841 ORF NotI R	CGCGGCGGCCGCCATACTCTGGACGACGTTCGTGC
	AFriboB SpeI F	CGCGACTAGTCCCGGGCTGCAGGAATTCG
	AFriboB XbaI R	CGCGTCTAGACCCGGGCTGCAGGAATTCGATAAG
	thiAp XbaI F	CGCGTCTAGACGACCTGGCACCTACAGAAGAATC
	thiAp SpeI R	CGCGACTAGTGTTGACTCAGTTCAATGGTTCGAC
**pGEM *AN3841/sac1-(GA)X5-GFP::AFpyrG***	AN3841 ORF ApaI F	CGCGGGGCCCCGTTTGCTCTCATTACTCGGAGGTC
	AN3841 ORFns SpeI R	CGCGACTAGTCTCGATCCTGGTCTTTCCTTCTTC
	AN3841 3 SpeI F	CGCGACTAGTCGTATTTCCTGCACTCATACCTGC
	AN3841 3 NotI R	CGCGGCGGCCGCCTTCTTAAGGACTTCTCCAGCAGTG
	GAGFP SpeI F	GCGACTAGTGGAGCTGGTGCAGGCGCTGGA
	AFpyrG SpeI R	CGGACTAGTCTGTCTGACAGGAGGCACTGATGCG
**pGEM::AFpyroA AlcAp/AN8672**	AN8672_5_ApaIF	CGCGGGGCCCGTGGTACTACGTAGAACCTCTAGAAG
	AN8672_5_SpeIR	CGCGACTAGTGGCAGGAATGAGAAAGTGAGGAAC
	AN8672_ORF_SpeIF	CGCGACTAGTATGGCTTCAGATAGCCTCCACCCG
	AN8672_ORF_NotIR	CGCGGCGGCCGCGATCTTTGTCTCAACGCCGGTAAAG
	alcA_SpeIR	CGCGACTAGTATTTTGAGGCGAGGTGATAGG
	AFpyrG SpeI F	CGCGACTAGTGCCTCAAACAATGCTCTTCACCC

Supplementary Table S4: Phylogenetic tree IDs

Accession	Entry Name	Species	Clade
**O60119**	SCS2_SCHPO	*Schizosaccharomyces pombe*	Fungi
**Q10484**	SCS22_SCHPO	*Schizosaccharomyces pombe*	Fungi
**A0A2K0UIM3**	A0A2K0UIM3_TRIHZ	*Trichoderma harzianum*	Fungi
**A0A0F9XAZ7**	A0A0F9XAZ7_TRIHZ	*Trichoderma harzianum*	Fungi
**B4F9V2**	B4F9V2_ZEA	*Zea mays*	Plants
**A0A1S3X8G7**	A0A1S3X8G7_NICTA	*Nicotiana tabacum*	Plants
**Q01IM1**	Q01IM1_ORYSA	*Oryza sativa*	Plants
**A0A178VEY8**	A0A178VEY8_ARATH	*Arabidopsis thaliana*	Plants
**A0A178WBB7**	A0A178WBB7_ARATH	*Arabidopsis thaliana*	Plants
**Q6DGS7**	VapA_DANRE	*Danio rerio*	Animals
**Q6P2B0**	VapB_DANRE	*Danio rerio*	Animals
**Q9WV55**	VAPA_MOUSE	*Mus musculus*	Animals
**Q9QY76**	VAPB_MOUSE	*Mus musculus*	Animals
**P40075**	SCS2_YEAST	*Saccharomyces cerevisiae*	Fungi
**Q6Q595**	SCS22_YEAST	*Saccharomyces cerevisiae*	Fungi
**Q5B4X4/ AN4406**	Q5B4X4_EMENI	*Aspergillus nidulans*	Fungi
**A0A5C3E0L5**	A0A5C3E0L5_USTRI	*Ustilago trichophora*	Fungi
**O95292**	VAPB_HUMAN	*Homo sapiens*	Animals
**Q9P0L0**	VAPA_HUMAN	*Homo sapiens*	Animals
**Q871I7**	Q871I7_NEUCS	*Neurospora crassa*	Fungi
**O44782**	O44782_CAEEL	*Caenorhabditis elegans*	Animals
**Q7KVX5**	Q7KVX5_DROME	*Drosophila melanogaster*	Animals
**Q9W4N8**	Q9W4N8_DROME	*Drosophila melanogaster*	Animals

Supplementary Data

Data_Figure 4:

**Calcofluor Fluorescence intensity**				
**Distance Bin (µm)**	**Group**	**Test Used**	**p-value**	**Significant (p < 0.05)**
**0–1**	**WT vs ΔvapA**	**Mann-Whitney U test**	**0.1998**	**No**
**1–2**	**WT vs ΔvapA**	**Unpaired t-test**	**2.64E-09**	**Yes**
**2–3**	**WT vs ΔvapA**	**Unpaired t-test**	**0.0764**	**No**
**3–4**	**WT vs ΔvapA**	**Welch's t-test**	**0.000501**	**Yes**
**4–5**	**WT vs ΔvapA**	**Welch's t-test**	**2.48E-11**	**Yes**
**5–6**	**WT vs ΔvapA**	**Mann-Whitney U test**	**0.0000366**	**Yes**
**6–7**	**WT vs ΔvapA**	**Welch's t-test**	**5.67E-09**	**Yes**
**7–8**	**WT vs ΔvapA**	**Welch's t-test**	**0.00638**	**Yes**
**8–9**	**WT vs ΔvapA**	**Welch's t-test**	**0.000026**	**Yes**
**9–10**	**WT vs ΔvapA**	**Mann-Whitney U test**	**0.0000165**	**Yes**

**$PH^{plc\delta}$ fluorescence intensity**				
**Distance Bin**	**Group**	**Test Used**	**p-value**	**Significant (p < 0.05)**
**0-1 µm**	**WT vs ΔvapA**	**Mann-Whitney U test**	**1.65E-05**	**Yes**
**1-2 µm**	**WT vs ΔvapA**	**Unpaired t-test**	**1.984E-16**	**Yes**
**2-3 µm**	**WT vs ΔvapA**	**Unpaired t-test**	**2.225E-12**	**Yes**
**3-4 µm**	**WT vs ΔvapA**	**Unpaired t-test**	**1.054E-16**	**Yes**
**4-5 µm**	**WT vs ΔvapA**	**Unpaired t-test**	**2.284E-17**	**Yes**
**5-6 µm**	**WT vs ΔvapA**	**Unpaired t-test**	**2.769E-21**	**Yes**
**6-7 µm**	**WT vs ΔvapA**	**Unpaired t-test**	**1.007E-16**	**Yes**
**7-8 µm**	**WT vs ΔvapA**	**Unpaired t-test**	**2.171E-16**	**Yes**
**8-9 µm**	**WT vs ΔvapA**	**Welch's t-test**	**1.046E-07**	**Yes**

**9-10 µm**	**WT vs ΔvapA**	**Unpaired t-test**	**2.33E-10**	**Yes**

Data_Figure 5:

**DnfA-GFP**				
**Distance Bin (µm)**	**Comparison**	**Test Used**	**p-value**	**Significant**
**0-1**	**WT vs ΔvapA**	**Mann-Whitney U**	**0.035504**	**Yes**
**1-2**	**WT vs ΔvapA**	**Mann-Whitney U**	**3.66E-05**	**Yes**
**2-3**	**WT vs ΔvapA**	**Mann-Whitney U**	**0.024045**	**Yes**
**3-4**	**WT vs ΔvapA**	**Mann-Whitney U**	**7.66E-05**	**Yes**
**4-5**	**WT vs ΔvapA**	**Mann-Whitney U**	**0.00726**	**Yes**
**0-1**	**WT vs Δap2σ**	**Mann-Whitney U**	**0.004081**	**Yes**
**1-2**	**WT vs Δap2σ**	**Mann-Whitney U**	**3.66E-05**	**Yes**
**2-3**	**WT vs Δap2σ**	**Mann-Whitney U**	**1.65E-05**	**Yes**
**3-4**	**WT vs Δap2σ**	**Mann-Whitney U**	**3.66E-05**	**Yes**
**4-5**	**WT vs Δap2σ**	**Mann-Whitney U**	**0.001106**	**Yes**
**DnfB-GFP**				
**Distance Bin**	**Comparison**	**Test Used**	**p-value**	**Significant (p < 0.05)**
**0-1**	**WT vs ΔvapA**	**Mann-Whitney U test**	**0.031252**	**Yes**
**1-2**	**WT vs ΔvapA**	**Mann-Whitney U test**	**3.66E-05**	**Yes**
**2-3**	**WT vs ΔvapA**	**Mann-Whitney U test**	**1.65E-05**	**Yes**
**3-4**	**WT vs ΔvapA**	**Unpaired t-test**	**0.005957**	**Yes**
**4-5**	**WT vs ΔvapA**	**Unpaired t-test**	**5.85E-01**	**No**
**0-1**	**WT vs Δap2σ**	**Mann-Whitney U test**	**0.001759**	**Yes**
**1-2**	**WT vs Δap2σ**	**Mann-Whitney U test**	**3.66E-05**	**Yes**
**2-3**	**WT vs Δap2σ**	**Mann-Whitney U test**	**1.65E-05**	**Yes**
**3-4**	**WT vs Δap2σ**	**Unpaired t-test**	**1.76E-05**	**Yes**
**4-5**	**WT vs Δap2σ**	**Welch's t-test**	**1.04E-12**	**Yes**
**GFP-ChsB**				
**Distance Bin**	**Comparison**	**Test Used**	**p-value**	**Significant (p < 0.05)**

**0-1**	**WT vs ΔvapA**	**Mann-Whitney U test**	**0.644412**	**No**
**1-2**	**WT vs ΔvapA**	**Mann-Whitney U test**	**0.035089**	**Yes**
**2-3**	**WT vs ΔvapA**	**Mann-Whitney U test**	**0.000591**	**Yes**
**3-4**	**WT vs ΔvapA**	**Unpaired t-test**	**0.000152**	**Yes**
**4-5**	**WT vs ΔvapA**	**Unpaired t-test**	**8.83E-09**	**Yes**
**0-1**	**WT vs Δap2σ**	**Mann-Whitney U test**	**0.918309**	**No**
**1-2**	**WT vs Δap2σ**	**Unpaired t-test**	**0.138674**	**No**
**2-3**	**WT vs Δap2σ**	**Welch's t-test**	**0.000239**	**Yes**
**3-4**	**WT vs Δap2σ**	**Mann-Whitney U test**	**0.060602**	**No**
**4-5**	**WT vs Δap2σ**	**Mann-Whitney U test**	**0.000858**	**Yes**
**wt vs ΔvapA Ap2σ-mRFP**				
**Distance Bin (µm)**	**Compasion**	**Test Used**	**p-value**	**Significant**
**0-1 µm**	**WT vs ΔvapA**	**Mann-Whitney U**	**5.09E-05**	**Yes**
**1-2 µm**	**WT vs ΔvapA**	***t*-test**	**2.18E-06**	**Yes**
**2-3 µm**	**WT vs ΔvapA**	***t*-test**	**2.72E-06**	**Yes**
**3-4 µm**	**WT vs ΔvapA**	***t*-test**	**0.017202**	**Yes**
**4-5 µm**	**WT vs ΔvapA**	**Mann-Whitney U**	**0.015941**	**Yes**
**5-6 µm**	**WT vs ΔvapA**	***t*-test**	**1.22E-11**	**Yes**
**6-7 µm**	**WT vs ΔvapA**	***t*-test**	**0.061274**	**No**
**7-8 µm**	**WT vs ΔvapA**	**Welch's *t*-test**	**0.002453**	**Yes**
**8-9 µm**	**WT vs ΔvapA**	**Welch's *t*-test**	**0.270866**	**No**
**9-10 µm**	**WT vs ΔvapA**	**Mann-Whitney U**	**0.007937**	**Yes**
**GFP-SynA or mCH-SynA**				
**Distance Bin**	**Test Used**	**p-value**	**Significant**	
**0-1**	**WT vs ΔvapA**	**1.65E-05**	**Yes**	
**1-2**	**WT vs ΔvapA**	**3.66E-05**	**Yes**	
**2-3**	**WT vs ΔvapA**	**1.65E-05**	**Yes**	
**3-4**	**WT vs ΔvapA**	**3.66E-05**	**Yes**	
**4-5**	**WT vs ΔvapA**	**2.59E-07**	**Yes**	
**0-1**	**WT vs Δap2σ**	**0.631542**	**No**	
**1-2**	**WT vs Δap2σ**	**5.82E-12**	**Yes**	
**2-3**	**WT vs Δap2σ**	**2.83E-11**	**Yes**	
**3-4**	**WT vs Δap2σ**	**1.28E-13**	**Yes**	
**4-5**	**WT vs Δap2σ**	**1.65E-05**	**Yes**	

Data_Figure 6:

Filipin III fluorescence intensity profile				
Distance Bin (Î¼m)	Comparison	Test Used	p-value	Significant ($p < 0.05$)
0-1	WT vsΔap2	Mann-Whitney U	1.65E-05	Yes
0-1	WT vs ΔvapA	Mann-Whitney U	0.199825	No
1-2 µm	WT vsΔap2	Mann-Whitney U	3.66E-05	Yes
1-2 µm	WT vs ΔvapA	Mann-Whitney U	3.66E-05	Yes
2-3 µm	WT vsΔap2	Mann-Whitney U	2.61E-05	Yes
2-3 µm	WT vs ΔvapA	Mann-Whitney U	1.65E-05	Yes
3-4 µm	WT vsΔap2	Mann-Whitney U	0.214494	No
3-4 µm	WT vs ΔvapA	Mann-Whitney U	3.66E-05	Yes
4-5 µm	WT vsΔap2	Mann-Whitney U	1.65E-05	Yes
4-5 µm	WT vs ΔvapA	Mann-Whitney U	1.65E-05	Yes
5-6 µm	WT vsΔap2	Mann-Whitney U	0.000901	Yes
5-6 µm	WT vs ΔvapA	Mann-Whitney U	3.66E-05	Yes
6-7 µm	WT vsΔap2	Mann-Whitney U	0.001106	Yes
6-7 µm	WT vs ΔvapA	Mann-Whitney U	3.66E-05	Yes
7-8 µm	WT vsΔap2	Mann-Whitney U	0.000412	Yes
7-8 µm	WT vs ΔvapA	Mann-Whitney U	0.000412	Yes

Data_Figure 7:

SlaB-GFP cortical fluorescence					
wt vs ΔvapA					
**Distance Bin (1µm)**	**Test Used**	**WT Mean**	**ΔvapA Mean**	**P-value**	**Significant**
0-1 µm	Mann-Whitney U test	4080.87	3335.374	0.472789	No
1-2 µm	Mann-Whitney U test	4329.113	3151.293	0.140955	No
2-3 µm	Welch's *t*-test	4612.937	2952.585	0.00784	Yes
3-4 µm	Welch's *t*-test	6985.324	5433.457	0.039661	Yes
4-5 µm	Welch's *t*-test	8082.514	5422.085	0.00104	Yes
5-6 µm	Welch's *t*-test	9591.878	7731.154	0.092897	No

**6-7 µm**	**Welch's *t*-test**	**9213.148**	**11975.03**	**0.005338**	**Yes**
**7-8 µm**	**Welch's *t*-test**	**8407.11**	**9583.851**	**0.097568**	**No**
**8-9 µm**	**Mann-Whitney U test**	**10451.09**	**6902.15**	**0.000308**	**Yes**
**9-10 µm**	**Welch's *t*-test**	**5818.072**	**5065.725**	**0.255285**	**No**
**wt vs Δap2σ**					
**Distance Bin (1µm)**	**Test Used**	**WT Mean**	**Δap2σ Mean**	**P-value**	**Significant**
**0-1 µm**	**Welch's *t*-test**	**4080.87**	**3845.005**	**0.65927**	**No**
**1-2 µm**	**Welch's *t*-test**	**4329.113**	**3193.884**	**0.025339**	**Yes**
**2-3 µm**	**Welch's *t*-test**	**4612.937**	**2958.951**	**0.000278**	**Yes**
**3-4 µm**	**Mann-Whitney U test**	**6985.324**	**4136.268**	**0.000592**	**Yes**
**4-5 µm**	**Welch's *t*-test**	**8082.514**	**4889.978**	**3.69E-05**	**Yes**
**5-6 µm**	**Welch's *t*-test**	**9591.878**	**5895.083**	**0.00432**	**Yes**
**6-7 µm**	**Welch's *t*-test**	**9213.148**	**6149.946**	**0.007211**	**Yes**
**7-8 µm**	**Welch's *t*-test**	**8407.11**	**9518.25**	**0.103874**	**No**
**8-9 µm**	**Welch's *t*-test**	**10451.09**	**7585.106**	**7.18E-05**	**Yes**
**9-10 µm**	**Welch's *t*-test**	**5818.072**	**9395.107**	**3.87E-05**	**Yes**
**SagA-GFP cortical fluorescence**					
WT vs Δap2σ					
**Distance Bin (1µm)**	**Test Used**	**WT Mean**	**Î”ap2Ïƒ Mean**	**P-value**	**Significant?**
**0-1 µm**	**Mann-Whitney U test**	**995.828**	**893.5919**	**0.837472**	**No**
**1-2 µm**	**Welch's *t*-test**	**1778.026**	**916.3829**	**0.007862**	**Yes**
**2-3 µm**	**Welch's *t*-test**	**1002.268**	**1942.667**	**0.000493**	**Yes**
**3-4 µm**	**Welch's *t*-test**	**1744.309**	**934.543**	**0.009881**	**Yes**
**4-5 µm**	**Mann-Whitney U test**	**2778.076**	**1190.538**	**0.008913**	**Yes**
**5-6 µm**	**Welch's *t*-test**	**4346.904**	**1613.165**	**2.28E-06**	**Yes**
**6-7 µm**	**Welch's *t*-test**	**4471.907**	**1932.429**	**0.000863**	**Yes**
**7-8 µm**	**Mann-Whitney U test**	**3283.894**	**2000.241**	**0.090587**	**No**
**8-9 µm**	**Welch's *t*-test**	**4395.416**	**2584.445**	**5.19E-05**	**Yes**
**9-10 µm**	**Mann-Whitney U test**	**2185.66**	**1900.356**	**0.238204**	**No**
**WT vs ΔvapA**					
**Distance Bin (1µm)**	**Test Used**	**WT Mean**	**Î”scs2 Mean**	**P-value**	**Significant?**
**0-1 µm**	**Welch's *t*-test**	**995.828**	**1435.52**	**0.11021**	**No**
**1-2 µm**	**Welch's *t*-test**	**1778.026**	**1688.486**	**0.79709**	**No**
**2-3 µm**	**Welch's *t*-test**	**1002.268**	**1494.827**	**0.014525**	**Yes**
**3-4 µm**	**Welch's *t*-test**	**1744.309**	**1173.13**	**0.077105**	**No**
**4-5 µm**	**Mann-Whitney U test**	**2778.076**	**1422.309**	**0.035504**	**Yes**
**5-6 µm**	**Welch's *t*-test**	**4346.904**	**2517.361**	**0.00051**	**Yes**
**6-7 µm**	**Welch's *t*-test**	**4471.907**	**2390.719**	**0.003856**	**Yes**
**7-8 µm**	**Mann-Whitney U test**	**3283.894**	**2476.014**	**0.472789**	**No**
**8-9 µm**	**Welch's *t*-test**	**4395.416**	**2879.238**	**0.000295**	**Yes**

**9-10 µm**	**Welch's *t*-test**	**2185.66**	**3115.981**	**0.001352**	**Yes**
**MyoA-GFP cortical fluorescence**					
**wt vs Δap2σ**					
**Distance Bin (µm)**	**Test Used**	**WT Mean**	**Δap2σ Mean**	**P-value**	**Significant ?**
**1-2 µm**	**Welch's *t*-test**	**9853.497**	**13935.44**	**0.051453**	**No**
**2-3 µm**	**Welch's *t*-test**	**20622.6**	**16153**	**0.133545**	**No**
**3-4 µm**	**Welch's *t*-test**	**24814.18**	**15930.5**	**0.001309**	**Yes**
**4-5 µm**	**Welch's *t*-test**	**49947.35**	**31494.67**	**0.000165**	**Yes**
**5-6 µm**	**Welch's *t*-test**	**25485.63**	**33806.4**	**0.022127**	**Yes**
**6-7 µm**	**Welch's *t*-test**	**41886**	**28402.1**	**1.83E-07**	**Yes**
**7-8 µm**	**Welch's *t*-test**	**28790.53**	**19790.07**	**0.001992**	**Yes**
**8-9 µm**	**Welch's *t*-test**	**12721.73**	**17570.33**	**0.006602**	**Yes**
**9-10 µm**	**Mann-Whitney U test**	**8047.328**	**13991.8**	**0.130968**	**No**
**wt vs ΔvapA**					
**Distance Bin (µm)**	**Test Used**	**WT Mean**	**ΔvapA Mean**	**P-value**	**Significant ?**
**1-2 µm**	**Welch's *t*-test**	**9853.497**	**8915.042**	**0.586204**	**No**
**2-3 µm**	**Welch's *t*-test**	**20622.6**	**7212.13**	**0.000191**	**Yes**
**3-4 µm**	**Welch's *t*-test**	**24814.18**	**20655.53**	**0.145254**	**No**
**4-5 µm**	**Welch's *t*-test**	**49947.35**	**38486.61**	**0.004051**	**Yes**
**5-6 µm**	**Welch's *t*-test**	**25485.63**	**31302.06**	**0.020582**	**Yes**
**6-7 µm**	**Welch's *t*-test**	**41886**	**32446.46**	**4.26E-05**	**Yes**
**7-8 µm**	**Welch's *t*-test**	**28790.53**	**22543.78**	**0.046001**	**Yes**
**8-9 µm**	**Welch's *t*-test**	**12721.73**	**10774.76**	**0.165908**	**No**
**9-10 µm**	**Mann-Whitney U test**	**8047.328**	**5906.34**	**0.030239**	**Yes**
**AbpA-DsRed cortical fluorescence**					
**WT vs vapA**					
**Distance Bin**	**Test Used**	**p-value**	**Significant**		
**0-1 µm**	**Unpaired *t*-test**	**0.615678**	**No**		
**1-2 µm**	**Unpaired *t*-test**	**0.152373**	**No**		
**2-3 µm**	**Unpaired *t*-test**	**0.599123**	**No**		
**3-4 µm**	**Unpaired t-test**	**0.011679**	**Yes**		
**4-5 µm**	**Welch's *t*-test**	**1.99E-05**	**Yes**		
**5-6 µm**	**Mann-Whitney U test**	**0.260236**	**No**		
**6-7 µm**	**Mann-Whitney U test**	**3.66E-05**	**Yes**		
**7-8 µm**	**Unpaired *t*-test**	**5.27E-05**	**Yes**		
**8-9 µm**	**Unpaired *t*-test**	**5.53E-05**	**Yes**		
**9-10 µm**	**Mann-Whitney U test (n<3)**	**0.333333**	**No**		
**WT vs ap2**					

Distance Bin	Test Used	p-value	Significant		
**0-1 µm**	**Mann-Whitney U test**	**0.015941**	**Yes**		
**1-2 µm**	**Unpaired *t*-test**	**0.053626**	**No**		
**2-3 µm**	**Unpaired *t*-test**	**5.94E-08**	**Yes**		
**3-4 µm**	**Unpaired *t*-test**	**2.26E-06**	**Yes**		
**4-5 µm**	**Unpaired *t*-test**	**0.097221**	**No**		
**5-6 µm**	**Welch's *t*-test**	**0.000379**	**Yes**		
**6-7 µm**	**Mann-Whitney U test**	**3.66E-05**	**Yes**		
**7-8 µm**	**Unpaired *t*-test**	**0.020133**	**Yes**		
**8-9 µm**	**Unpaired *t*-test**	**0.095652**	**No**		
**9-10 µm**	**Mann-Whitney U test (n<3)**	**0.333333**	**No**		

Supplemental Figure S1

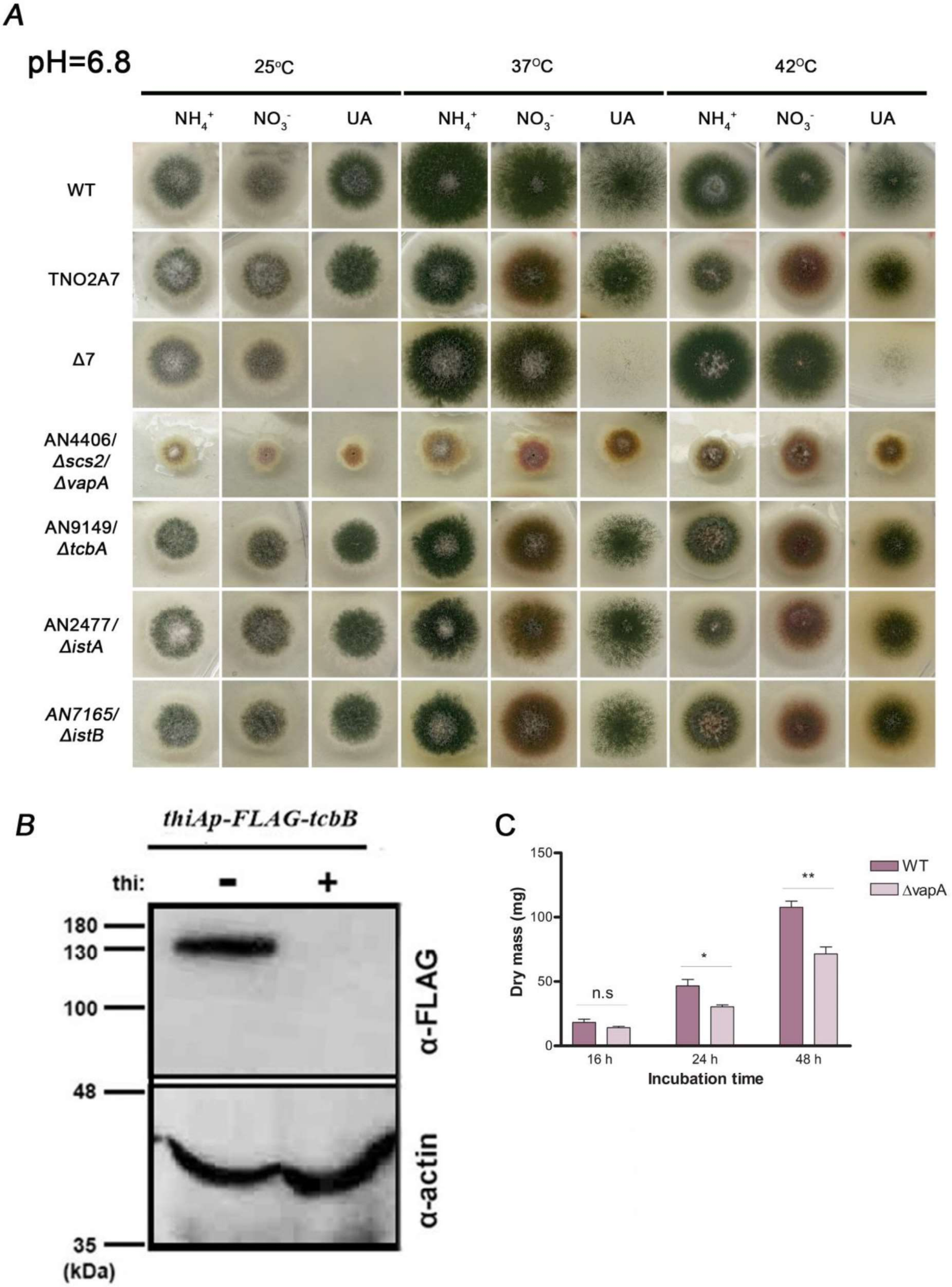

Supplemental Figure S2

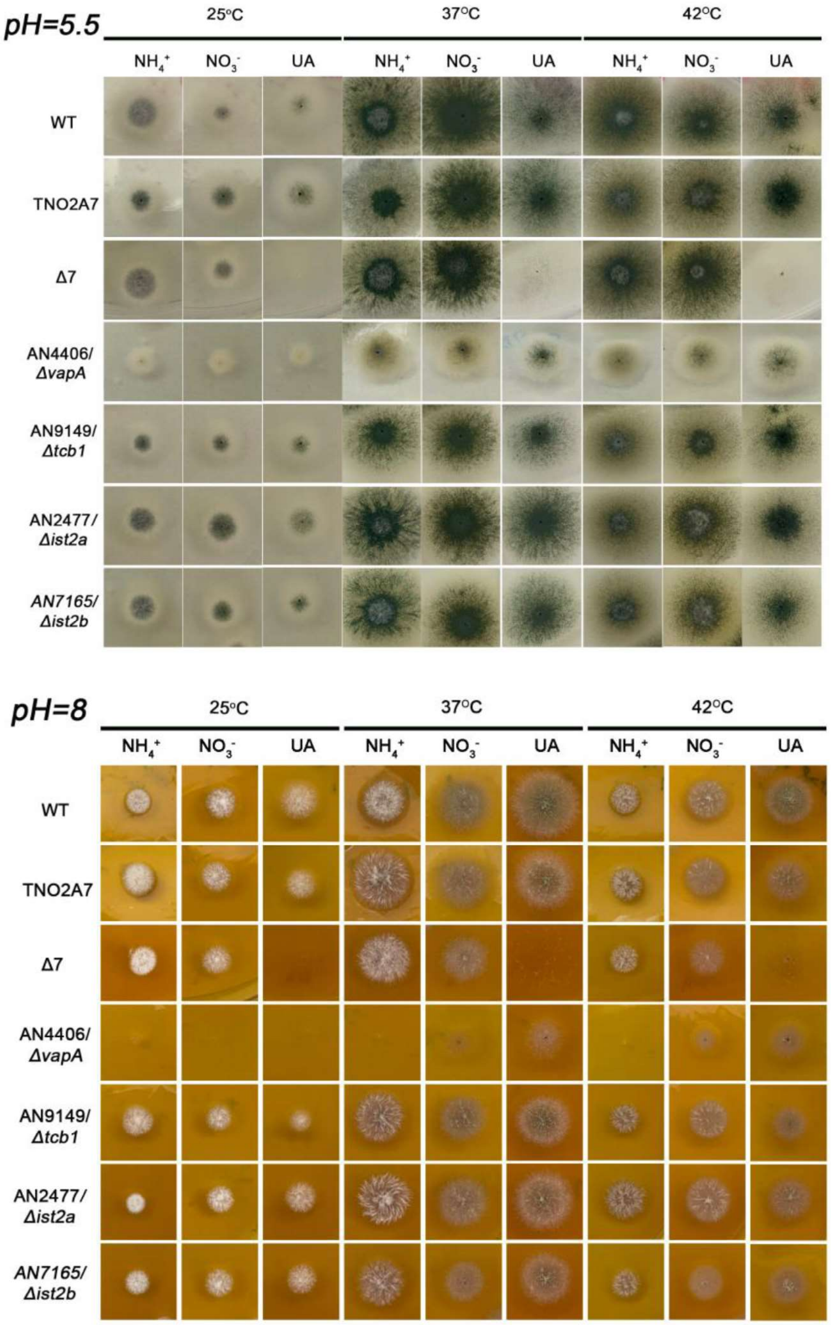

Supplemental Figure S3

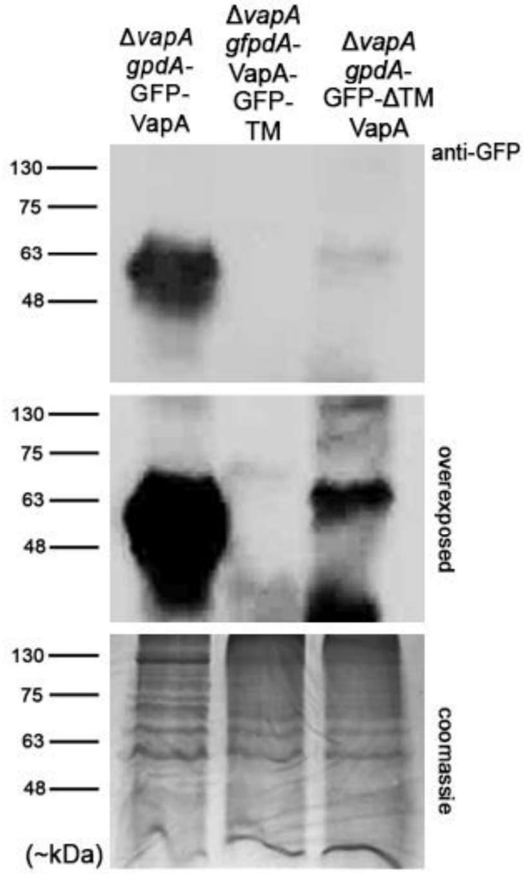

Suppelmental Figure S4

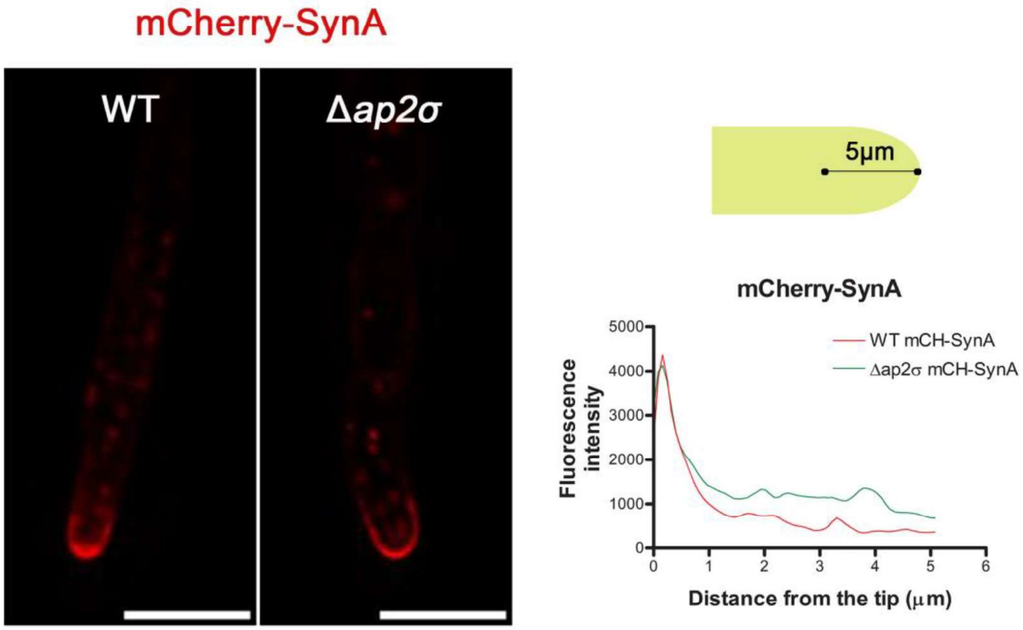

Supplemental Figure S5

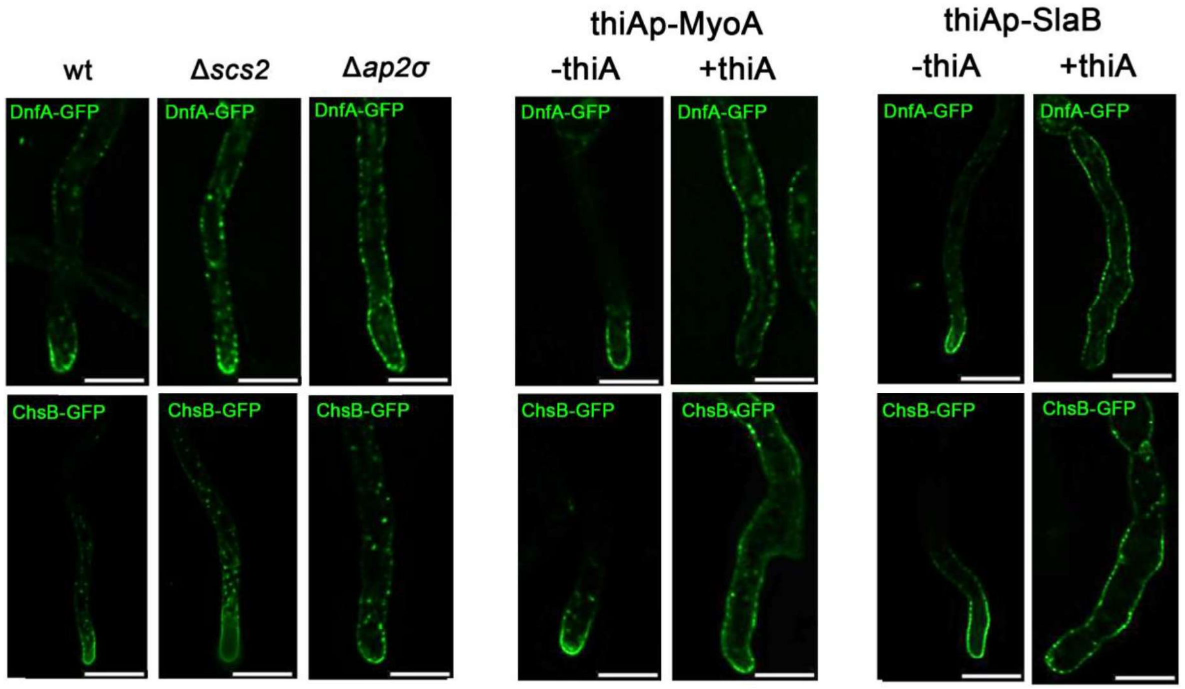

Supplement: Supplementary file 1 [file mic-13-063-s01.pdf]
